# The comparative advantages of HSR, AIR, and HA on the coordinated regional economic development in China

**DOI:** 10.1371/journal.pone.0338500

**Published:** 2026-01-05

**Authors:** Guo Dong Li, Wenshan Liu, You Zhi Xue, Sangbing Tsai

**Affiliations:** 1 School of Economics and Management, Civil Aviation University of China, Tianjin, China; 2 School of Economics, Nankai University, Tianjin, China; 3 Business School, Nankai University, Tianjin, China; 4 International Engineering and Technology Institute, Hong Kong, China; University of Washington, UNITED STATES OF AMERICA

## Abstract

This study aims to systematically compare the roles of high-speed rail (HSR), air transport (AIR), and their synergy in promoting coordinated regional economic development across different spatial scales in China. While the existing literature has primarily focused on individual transportation modes, the comparative advantages and synergistic effects of HSR and AIR on coordinated regional economic development remain understudied. To address this gap, we employ multi-dimensional panel data from 2008 to 2023, covering macro-regional, provincial, and city networks, to reveal the heterogeneous impacts and collaborative potential of these transportation networks. Our objective is to determine the optimal spatial scale for each transport mode and their collaboration to maximize coordinated regional economic development. Results demonstrate that AIR significantly enhances coordinated regional economic development at the macro-regional scale, HSR-AIR collaboration development markedly promotes coordinated regional economic development at the provincial scale, and HSR significantly strengthens coordinated regional economic development at the city scale. The dose-response model regression results further indicate that the positive effect of AIR networks on coordinated regional economic development exhibits stable enhancement with AIR development at the macro-regional scale; HSR-AIR collaboration’s promotive effect increases with its development at the provincial scale but encounters an upper growth limit; and at the city scale, HSR’s impact initially rises then declines, displaying diminishing marginal returns on coordinated regional economic development. Consequently, this study recommends formulating differentiated transport network planning policies tailored to specific spatial scales, leveraging the complementary advantages of HSR and AIR to foster coordinated regional economic development.

## 1. Introduction

Coordinated regional economic development (CRED) has always been the core of China’s regional development strategy, serving as a key policy framework for achieving balanced national economic and social development. As a persistent priority in economic and social development planning, CRED has been continuously deepened and strengthened [1]. According to China’s national development strategy, the Chinese government emphasized the importance of implementing the regional coordinated development strategy to address the imbalanced and inadequate regional economic development and achieve high-quality development. It is evident that CRED remains a critical issue for China in its ongoing economic transition and efforts to establish a more sustainable and balanced economic structure.

Under the impetus of a series of regional coordinated development strategies, China’s various regions have achieved rapid development. However, the problem of unbalanced and inadequate regional economic development remains prominent [2,3]. From the perspective of the GDP gap among China’s four major economic regions, the country’s economic development exhibits a trend of regional differentiation, with the problem of polarized development momentum becoming increasingly severe. Certain special regions also face significant development challenges [4]. The pace of structural optimization in the eastern region is faster than that of the western region, with better development quality and higher efficiency. The advantage of rapid development in the western region is gradually weakening, and the absolute gap in economic development levels is still widening. The northwest region, in particular, faces prominent pressures in resource and environmental protection, with a relatively weak development foundation and a slower pace of transformation and upgrading. The internal advantageous complementary industrial division system in the central region needs to be further optimized, and the cultivation of world-class urban agglomerations and central cities is yet to be strengthened. The economic development speed of the northeastern region has been relatively low for many years, and the degree of ageing is high, leading to a lack of development momentum. Furthermore, underdeveloped regions, historically significant areas, ecologically degraded areas, resource-based cities, and old industrial bases still face numerous difficulties and bottlenecks in their development [5]. Therefore, the balance and coordination of regional economic development within China need to be further enhanced, and the development problems of various regions need to be gradually solved in the process of promoting CRED.

Given China’s vast territory and large population, the disparities in the basic conditions among its regions are unparalleled in the world, posing a daunting challenge in coordinating regional development [6]. In the future, as regional industrial structures converge, the distribution of critical industries and industrial chain links continues to concentrate, and local protectionism and regional barriers still exist to a certain extent, the problems of unbalanced and inadequate regional economic development will persist [7,8]. The spatial agglomeration of the economy can lead to regional inequalities in people’s living standards, which can easily breed social problems and undermine the national objective of balanced growth and equitable development. Therefore, CRED must be further promoted [9,10]. The formulation of regional policies depends on the real context and foundation of inter-regional transportation and economic differences [11]. However, the existing literature provides insufficient guidance for such differentiated policymaking. While the impacts of transportation infrastructure on regional development are widely acknowledged, comparative studies that systematically dissect the distinct roles of HSR and AIR across different spatial scales are remarkably scarce. More critically, the potential synergistic effect of their coordinated development on CRED remains largely unexplored. Consequently, it is necessary to investigate the impact of transportation networks on CRED at different spatial scales, which will provide a basis for the formulation of relevant regional policies. As the economy enters a new stage of development, in the context of increasingly networked regional economic linkages, actively exploring the role of transportation networks in promoting CRED holds profound theoretical and practical significance for driving the sustainable, coordinated, healthy, and high-quality development of the economy and society [12].

**Fig 1:**
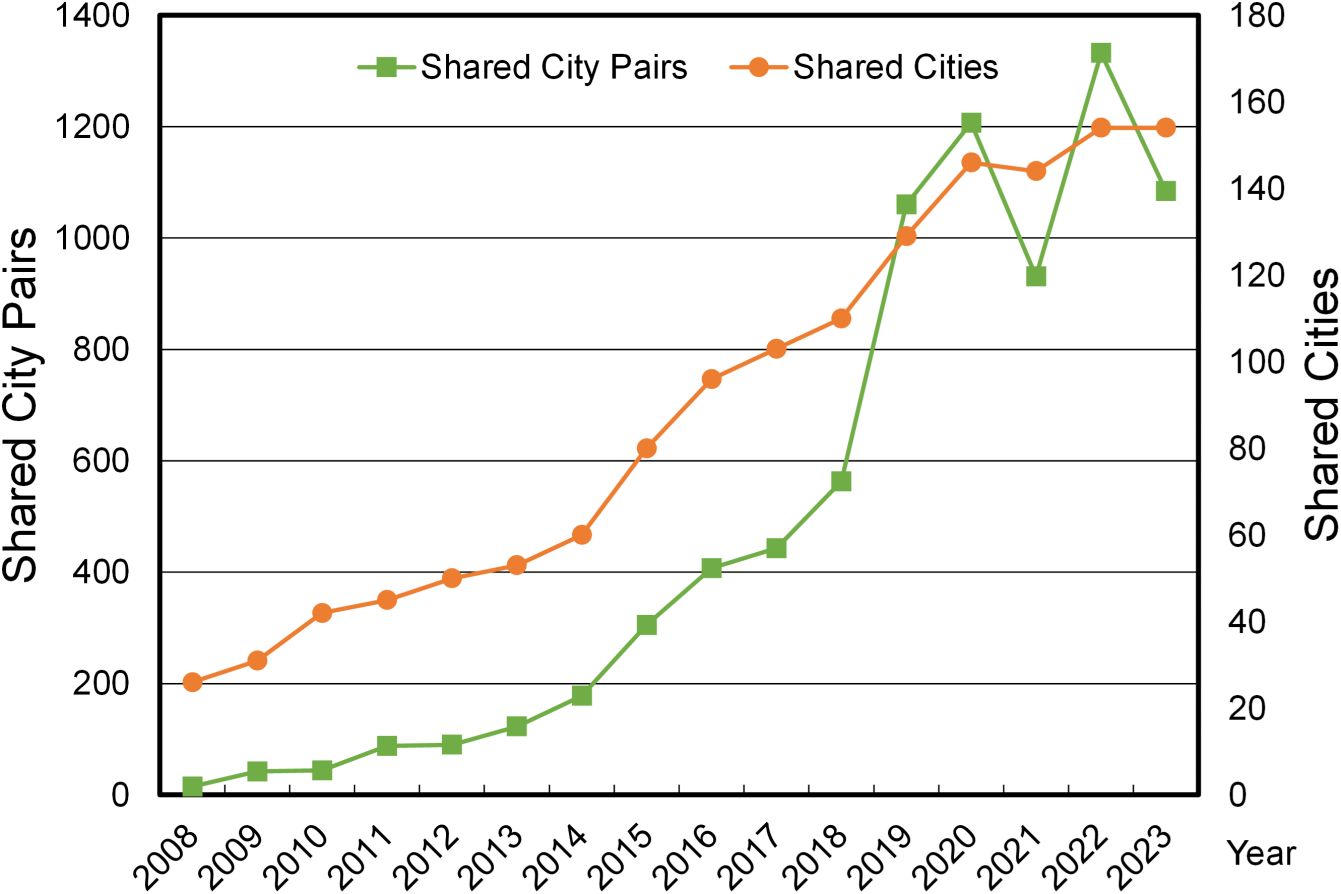
The scale of cities and city pairs shared by China’s HSR and AIR networks from 2008 to 2023.

Since the opening of the Beijing-Tianjin intercity train in 2008, the scale of China’s high-speed rail (HSR) network has grown rapidly, and its coverage has gradually expanded [13]. Given the comparability of HSR and AIR in terms of speed, price, comfort, and safety, as well as the similarity in their target passenger groups, the spatial coverage of the HSR and AIR networks has become increasingly convergent, with the overlapping degree continuously increasing and the overlapping scope becoming larger and larger [14]. This has led to a certain degree of overlapping infrastructure investment and suboptimal resource allocation. As shown in Fig 1, since 2008, the number of cities and city pairs in the competitive network between China’s HSR and AIR has increased from 26 and 15 in 2008154 and 1,084 in 2023, respectively, with the scale of the competitive network continuously expanding. Meanwhile, due to the obvious advantages of HSR over AIR in terms of door-to-door time, punctuality rate, frequency, and other passenger service characteristics, HSR has stronger appeal to short- and medium-haul passengers, leading to fierce competition with the AIR industry [15]. Therefore, how to explore the differentiated development paths of HSR and AIR and achieve their complementary advantages and coordinated development is an urgent problem to be solved. In this regard, exploring the differences in the CRED effects of HSR and AIR presents a new direction for investigating the complementary advantages and coordinated development of HSR and AIR [16].

Previous research on the impact of transportation on CRED has focused more on HSR or transportation as a whole, while research on the AIR sector has been relatively scarce. As China is committed to building a comprehensive transportation system, with AIR transport being an important component, its CRED effects cannot be ignored, and the collaboration between HSR and AIR in this regard also needs further investigation [16]. The civil aviation industry has the characteristics of high transportation efficiency and strong driving ability for peripheral regions, which may also make it influential in promoting CRED [17]. Meanwhile, due to the different transportation characteristics and network features, the impacts of HSR and AIR on CRED may differ in terms of space and scale [17–21]. Spatially, the transportation characteristics and network structure of AIR make it more conducive to driving the development of regions such as the western areas, which have large areas, relatively sparse population distribution, and uneven urban development, aligning with the demand for coordinated development between China’s peripheral and core regions. In contrast, HSR is more suitable for meeting the rapid transportation needs between the economically connected and relatively densely populated eastern and central regions [17,19,20,22]. In terms of scale, AIR is a more economically efficient transportation mode for fast connections between distant regions, which fits the coordinated development between regions and provinces, while HSR has advantages in enhancing the economic linkages between central cities and their relatively close rural areas, thereby better promoting the coordinated development between central and non-central cities and between urban and rural areas at the urban agglomeration scale [17,23,24]. Therefore, comparing the heterogeneous comparative advantages of HSR and AIR networks in promoting CRED and examining whether the collaboration between HSR and AIR can promote CRED hold great research significance and practical value.

In light of this research gap, this study aims to systematically investigate and compare the roles of HSR, AIR, and their synergy in fostering CRED in China. Our primary objectives are threefold: (1) To quantify and compare the heterogeneous impacts of HSR and AIR network development on CRED at three distinct spatial scales: the macro-regional, provincial, and city levels. (2) To empirically test whether the synergistic development of HSR-AIR collaboration generates a significant positive effect on CRED, and to identify the spatial scale at which this synergy is most potent. (3) To uncover the dynamic evolution of these impacts by employing a Dose-Response (DR) model, which reveals how the influence of HSR, AIR, and HSR-AIR collaboration on CRED changes with their respective levels of development. By achieving these objectives, this study seeks to move beyond the monolithic treatment of transport infrastructure and provide a scalable, spatially sensitive framework for optimizing transport network planning to achieve balanced regional growth.

To reveal the impacts of HSR and AIR on CRED between different dimensional “regional pairs,” we first collected data on HSR and AIR operations, as well as regional economic statistics, from 2008 to 2023 for 7 macro-regions, 36 provinces or regions, and 366 prefecture-level administrative areas in China. This allowed us to construct three panels: a macro-regional dimension network panel with 21 “macro-region pairs”, a provincial dimension network panel with 465 “provincial pairs”, and a city dimension network panel with 2,540 “city pairs”. Secondly, this study constructed indicators and measured the levels of CRED in China, AIR network development (AIR), HSR network development (HSR), and HSR-AIR collaboration *(*HA*)*. We then conducted a descriptive analysis of the temporal and spatial heterogeneity of the above research subjects, obtaining the development trajectories and current status, thereby observing the potential impact patterns and possible heterogeneous manifestations of AIR, HSR, and HSR-AIR collaboration on CRED. Thirdly, we used OLS models and DR models to analyze the impacts of AIR, HSR, and HSR-AIR collaboration on CRED, obtaining the following empirical results: baseline model regression results, DR model verification results, and robustness check results. We then analyzed these test results in depth. Finally, this study summarized the main research conclusions and provided policy recommendations based on them, demonstrating the practical significance of the research findings.

The unique academic value of this study mainly lies in the following four aspects:

First, this study comprehensively compared the impacts of HSR and AIR networks on CRED. Existing research has largely focused on a single transportation mode, while comparative analyses of the two have been scarce. This study systematically compared the similarities and differences between HSR and AIR in promoting CRED from the spatial dimensions of macro-regions, provinces, and cities, thereby enriching the relevant research. Different transportation modes have their own technological characteristics and economic attributes, and their mechanisms of action on regional development differ. The comparative analysis in this study helps to deepen the understanding of the similarities and differences between different transportation networks in promoting regional coordinated development, providing theoretical support for formulating differentiated transportation infrastructure investment policies. This not only enriches the research findings in the relevant field but also provides a basis for the government to coordinate the development of different transportation modes.

Secondly, this study explored the impact of the synergistic development of HSR and AIR on CRED. Existing research has rarely focused on the impact of collaboration between the two transportation modes, despite the fact that there is a certain degree of complementarity between transportation modes, and synergistic development can generate synergistic effects, thereby better promoting the coordinated regional economy. This study found that at the provincial scale, the synergistic development of HSR and AIR has a more significant impact on CRED, providing a theoretical basis for promoting the integrated development of transportation modes and realizing their complementary advantages. This not only enriches the research on the relationship between transportation infrastructure and regional development but also provides a reference for the government in formulating policies for the construction of a comprehensive transportation system.

Thirdly, this study provided comprehensive empirical evidence of China’s CRED level, HSR network development level, and AIR network development level, and found that HSR and AIR networks exhibit significant dimensional heterogeneous effects in promoting CRED. Specifically, the HSR network is more effective in promoting CRED at the city and provincial dimensions, while the AIR network is more effective in promoting CRED at the macro-regional dimension. This finding provides a new perspective for a deeper understanding of the differentiated impact mechanisms of transportation infrastructure networks on CRED. The conclusions of this study provide a theoretical basis for formulating transportation infrastructure investment policies tailored to local conditions, which not only helps to better understand the complex relationship between transportation development and regional coordination but also lays a foundation for the government to formulate differentiated regional development strategies.

The other parts of this study are organized as follows. Section 2 reviews the relevant literature. Section 3 introduces empirical methods and describes the models and data statistics used in this paper. Section 4 presents the descriptive statistics and analysis of the spatial-temporal distribution characteristics of the main research subjects of this study. Section 5 conducts empirical tests, including baseline regression, DR model verification, and robustness checks. Section 6 presents the main research conclusions of this study and the policy recommendations based on them.

## 2. Literature review

### 2.1. Impact of rapid transportation networks on coordinated regional economic development (CRED)

The impact of rapid transit networks on CRED has long been a subject of academic debate, centered on a fundamental paradox: transportation improvements can foster balance through diffusion effects, yet may also exacerbate disparities via agglomeration effects. Existing literature reveals substantial divergences regarding the direction of impact, underlying mechanisms, and spatial heterogeneity.

#### 2.1.1. Debate on the direction of impact: convergence or divergence?.

A substantial body of research supports the role of rapid transit networks, particularly high-speed rail (HSR), in promoting CRED, underpinned by the diffusion effects arising from “time–space convergence.” From the perspective of new economic geography and using panel data from 2000–2014, Chen and Haynes (2017) found that HSR development significantly reduced the nationwide weighted coefficient of variation, Theil index, and Gini coefficient, thereby fostering regional economic convergence [25]. Wang et al. (2019), employing PSM-DID methods with matched data on HSR and listed companies from 2003–2016, observed that HSR operation led to a convergence trend in total factor productivity between firms in cities with and without HSR services, indicating significant diffusion effects [26]. Dai and Link (2025) further corroborate this view, finding that HSR services effectively reduced interregional inequality in GDP per capita and narrowed the wage gap between eastern and central China [27]. Through a polynomial inverse lag framework and a simultaneous equations system, Chen and Lu (2016) revealed a nonlinear negative correlation between post-reform economic growth and inequality in China, providing an earlier macro-level context for understanding the distributional effects of transport infrastructure [3].

However, the opposing “polarization effect” thesis is likewise supported by robust empirical evidence. Bian et al. (2018), leveraging data from 287 prefecture-level cities (2004–2014) and treating HSR opening as a quasi-natural experiment, found that HSR significantly widened regional economic disparities by facilitating factor mobility—an effect particularly pronounced in provincial capitals [28]. Spatial econometric analysis by Jin et al. (2020) uncovered a more complex picture that although HSR drove local economic growth, its spatial spillover effects were insignificant, and the growth dividends were primarily captured by large, developed cities, potentially exacerbating economic disparities in China [29]. A case study of the Yangtze River Delta by Ma et al. (2022) more directly concluded that HSR operation widened the economic development gap between central and peripheral cities. The mechanism lies in HSR accelerating the polarized flow of labor toward centers and reducing the investment attractiveness of smaller peripheral cities [30]. A case study of Jiangsu Province by Wei et al. (2020), using a multi-scale, multidimensional framework, revealed the evolution of regional inequality, spatial polarization, and place mobility. They found that the stability of the core–periphery structure reduces place mobility, explaining the inherent spatial logic sustaining polarization even when regional inequality ceases to worsen [6]. This polarization effect is also evident in the civil aviation sector. Yin Peiwei et al. (2022) found that the civil aviation industry has a positive impact on the agglomeration radiation effect of cities, but the role of large hub airports is stronger, potentially leading to further concentration of resources in core cities [24].

The root of this scholarly divergence lies in the nonlinear and context-dependent nature of rapid transit network impacts. Luo Fuzheng and Luo (2019), through theoretical modeling and empirical analysis, astutely observed that regional market agglomeration exhibits a negative polarization effect in the short run, which may transition toward a positive diffusion effect in the long run [31]. Similarly, the structural characteristics of the HSR network are crucial. Yi et al. (2021), applying complex network theory and a Spatial Durbin Model, found that the strength of spatial spillover effects varies among HSR hub cities with different topological properties [32]. Analyzing social networks, Lan and Zhang (2023) showed that while HSR networks enhance economic linkages within urban agglomerations, they also widen internal economic development disparities in most agglomerations, thereby inhibiting coordinated development. This suggests that a simplistic binary variable of “HSR connection” may obscure inherent inequitable effects embedded in the network structure [33]. Using the Herfindahl–Hirschman Index, Gini coefficient decomposition, kernel density estimation, and convergence models, Liu et al. (2022) revealed the polycentric economic spatial structure, significant regional disparities, and polarization characteristics of Chinese urban agglomerations. They also identified both absolute and conditional *β*-convergence, providing direct evidence of the complexity of network structure impacts—where polarization and convergence can coexist within the same system [7].

#### 2.1.2. Analysis of multidimensional mechanisms.

Rapid transit networks influence CRED through multiple micro- and macro-level channels, yet the efficiency and effectiveness of these channels remain subjects of debate.

From a micro-level perspective, accessibility serves as a core mediating variable. Transport infrastructure upgrades compress temporal and spatial distances, thereby altering locational advantages. Cao and Su (2024) found that transport infrastructure enhances regional resource allocation efficiency by promoting industrial agglomeration and mitigating market segmentation, while also generating positive spatial spillovers to neighboring areas. However, the benefits of such improved accessibility are not evenly distributed [11]. A study of the Yangtze River Delta by An et al. (2022) demonstrated that while HSR operation substantially improved overall connectivity within the urban network, it also exacerbated the imbalance in urban centrality, forming a hierarchical spatial structure centered on Shanghai, Nanjing, and Hangzhou, which in turn inhibited local economic growth in peripheral areas. This aligns with the classical proposition in the “core–periphery” model that enhanced connectivity may initially strengthen the position of core nodes [34]. Xu et al. (2021) developed a statistical framework using nighttime light data as a proxy for GDP; their study found that China’s regional development imbalances primarily stem from interregional rather than intraregional disparities, providing a new measurement tool for assessing the role of transport networks in addressing this primary contradiction of “interregional” disparity [4].

Factor mobility constitutes another key micro-level mechanism. Chen et al. (2022) found that infrastructure upgrades—specifically HSR and smart city initiatives—narrowed regional economic disparities by inducing labor mobility [35]. In contrast, Ma et al. (2022), focusing on the Yangtze River Delta, observed the opposite: HSR-induced labor mobility intensified the polarization of the periphery by the core [30]. This contradiction suggests that the direction of factor mobility—whether bidirectional diffusion or unidirectional agglomeration—depends on local conditions such as industrial base and public service levels in receiving areas [36]. Zheng et al. (2022) provided new evidence from the perspective of financial resource allocation: HSR access promoted the concentration of financial resources in connected cities. This effect was most pronounced in central China, followed by eastern China, and overall insignificant in the west. While conducive to narrowing intra-provincial and intra-regional disparities, it may nevertheless widen urban development gaps at the national level [37]. Yao and Zhao (2022) established a relationship between railway accessibility and urban economic growth, employing a mediation effects model to examine spatial spillovers and labor inflow effects. They found heterogeneous pathways through which accessibility operates: high-administrative-level large cities rely more on the indirect effects of labor inflows, deepening our understanding of the heterogeneity in factor mobility mechanisms [38]. Shioji (2001), in a study of internal migration in Japan, found that although migration carried higher human capital and slightly slowed regional convergence, the effect was minimal. This offers an international comparative perspective, suggesting that focusing solely on the quantity of factor mobility may be insufficient; attention must also be paid to its quality and structural impacts [39].

At the macro level, rapid transit networks profoundly reshape regional spatial patterns. They facilitate the evolution of urban agglomerations from monocentric structures toward polycentric, networked formations. In his doctoral dissertation, Xu (2023) systematically demonstrated, using panel data models and mediation effect models, that a polycentric spatial structure promotes CRED through three mechanisms: division of labor, sharing, and government intervention. However, his research also revealed that the impact of population polycentricity is significantly heterogeneous and requires context-specific approaches [36]. On the other hand, transport networks also influence regional specialization. A multi-region, multi-sector spatial economic model constructed by Pi and Wang (2025) indicates that HSR connection, by reducing trade costs, positively affects industries with initial comparative advantages and negatively affects those without, potentially reshaping regional industrial division of labor. The ultimate impact on coordinated development depends on the initial industrial endowment of each region [40]. Based on a Cobb–Douglas model and fixed-effects regression, Zeng et al. (2023) found that industrial agglomeration and a monocentric spatial structure positively influence urban agglomeration economic growth, supplementing our understanding of industrial agglomeration as a key pathway through which transport affects CRED [8]. Jin and Zhu (2023) focused specifically on the impact of HSR on industrial structure convergence/divergence, finding that HSR connection is positively correlated with the similarity of industrial structures between city pairs. However, this influence varies over time and differs across regions and industrial characteristics, providing new evidence in the debate on regional specialization versus industrial structural convergence [41].

#### 2.1.3. Transportation networks and labor market structural transformation.

Transport infrastructure, particularly HSR and AIR networks, serves as a key driver of coordinated regional economic development. By reshaping the spatial allocation of labor markets and industrial employment structures, it profoundly influences China’s ongoing transition from an agrarian economy toward one dominated by manufacturing and services. This transformation involves not only the reallocation of labor across sectors but also dynamic shifts in interregional development disparities. Theoretically, transport infrastructure—by reducing transportation costs, improving market accessibility, facilitating knowledge spillovers, and enhancing factor mobility—directly or indirectly guides the transfer of labor from low-productivity sectors (e.g., agriculture) to high-productivity sectors (e.g., manufacturing and services), thereby promoting industrial upgrading and regional economic convergence [25,42]. However, empirical studies reveal significant divergence regarding the actual effects of transport networks: some emphasize their positive role in accelerating structural transformation and regional convergence through labor mobility, while others point to their potential to exacerbate spatial polarization and employment imbalances, particularly within a core–periphery framework [34,43]. This dualistic relationship underscores the complexity of transport infrastructure’s impacts, necessitating critical analysis that incorporates institutional context, regional heterogeneity, and labor market characteristics.

From a theoretical perspective, transport infrastructure primarily influences labor market structural transformation through “labor mobility effects” and “industrial linkage effects.” First, transport networks reduce commuting and migration costs, enhance labor market integration, and enable workers to respond more flexibly to interregional wage differentials and employment opportunities [43]. For instance, HSR networks compress temporal and spatial distances, expand workers’ commuting radii and market potential, and facilitate labor shifts from agriculture to manufacturing and services—effects that are particularly pronounced when connecting core and peripheral cities [42,44]. Second, transport infrastructure alters industrial location choices, inducing enterprise agglomeration or dispersion, which in turn affects employment structures. For example, AIR networks tend to promote agglomeration in tradable services (e.g., finance and information technology), while HSR is more likely to stimulate growth in local service sectors such as retail and tourism [45,46]. Such industrial linkage effects are particularly salient in developing countries like China, where rapid transit networks accelerate the reallocation of production factors, driving “de-agriculturalization” and “re-industrialization” processes [47,48].

Nevertheless, empirical evidence indicates that the impact of transport infrastructure on labor market structural transformation exhibits significant spatial and sectoral heterogeneity—and at times, contradictory outcomes. On one hand, multiple studies support the view that transport networks promote structural transformation and regional convergence. In China, for example, HSR operation has significantly reduced labor misallocation through industrial upgrading and network connectivity mechanisms, leading to a decline in agricultural employment and a rise in service sector employment [42,43]. In Spain, HSR services accelerated the shift of labor from agriculture and manufacturing to services, reinforcing economic structural transformation [44]. These studies emphasize that transport infrastructure enhances labor allocation efficiency by expanding market potential and facilitating knowledge spillovers, thereby supporting coordinated regional economic development [27]. On the other hand, some literature suggests that transport networks may exacerbate regional inequality and employment polarization. In India, for instance, rural road construction prompted labor to exit agriculture but failed to significantly improve incomes or create new employment opportunities, highlighting the limitations of transport infrastructure in remote areas [49]. In the United States, highway expansion boosted service sector employment at the expense of manufacturing jobs, with negative spatial spillovers indicating that transport networks may lead to sectoral substitution rather than overall growth [50]. Within core–periphery structures, “siphoning effects” often benefit core cities while peripheral cities face employment losses. For example, in China’s Yangtze River Delta, HSR connectivity improved overall network connectivity but exacerbated imbalances in urban centrality, hindering local economic growth in peripheral areas [34,43].

These empirical discrepancies stem from various factors, including transport mode characteristics, regional development levels, and institutional environments. First, HSR and AIR influence labor markets through distinct mechanisms: HSR emphasizes short- to medium-distance commuting and intra-regional connectivity, whereas AIR serves long-distance and international markets, leading to differential impacts on employment structures [51, 52]. For example, in the Tokyo metropolitan area, inner-city airport accessibility correlates positively with labor productivity but not with employment density, suggesting that AIR networks rely more on agglomeration in high-end services [52]. In contrast, HSR in China more directly promotes employment in retail and hospitality, though its effects are constrained by competition from neighboring cities [46]. Second, regional heterogeneity significantly moderates the effects of transport networks. In rural areas of developing countries, road improvement projects have increased industrial employment and reduced agricultural employment, but these effects are often concentrated among women and urban regions, underscoring socioeconomic inequalities [48]. In China, HSR’s mitigating effect on labor misallocation is more pronounced in eastern regions and weaker in central and western regions, reflecting constraints imposed by initial endowments and institutional barriers [42,43]. Finally, policy interventions and infrastructure quality are also critical. In Indonesia, for instance, road maintenance investments yielded high benefit-cost ratios by increasing formal employment and incomes, but their effectiveness depended on local governance and funding allocation efficiency [53].

Within China’s unique economic transition context, rapid transit networks have accelerated labor market structural transformation while also revealing underlying contradictions. On one hand, HSR and AIR networks support the “new urbanization” and industrial upgrading strategies by facilitating cross-regional and cross-sectoral labor mobility. For example, HSR operation has significantly reduced labor misallocation through industrial rationalization and advancement, as well as enhanced urban connectivity, thereby improving overall labor productivity [42,54]. On the other hand, this transformation is not linear or balanced: HSR networks have created “agglomeration shadows” around core cities like Beijing and Shanghai, concentrating skill-intensive employment in the core while dispersing labor-intensive jobs to the periphery, thereby widening regional development gaps [33,43]. Moreover, although AIR networks have increased the employment share in tradable services, their contribution to overall employment growth remains limited, indicating a tendency toward specialization rather than inclusive development [45,55]. These contradictory perspectives reveal the dual-edged role of transport infrastructure in driving structural transformation—it can promote convergence through factor mobility yet may also reinforce polarization through market forces.

In summary, the relationship between transport networks and labor market structural transformation exhibits complex dialectics. Theoretically, transport infrastructure fosters economic transformation through labor mobility and industrial linkages; empirically, however, its effects are modulated by spatial, sectoral, and institutional factors, resulting in coexisting convergence and divergence. Future research should further integrate multi-dimensional data and examine how institutional innovations can optimize the benefits of transport networks. For China, coordinating HSR and AIR development, strengthening connectivity in peripheral regions, and improving labor market institutions will be crucial pathways toward achieving coordinated regional economic development.

#### 2.1.4. Rapid vs. traditional transport modes: a comparative perspective.

As representatives of rapid transport modes, high-speed rail (HSR) and aviation (AIR) differ significantly from traditional modes—such as road and conventional rail—in terms of speed, efficiency, accessibility, and regional economic impacts. This comparison extends beyond techno-economic characteristics to reveal the “double-edged sword” effect of rapid transport infrastructure: on one hand, HSR and AIR may foster coordinated regional development and factor mobility by compressing time-space distances; on the other, they risk reinforcing core-periphery structures and exacerbating interregional inequality. While traditional modes like road and conventional rail are relatively slower and less efficient, they may, under specific regional contexts, play a more balanced distributive role. Drawing on existing literature, this section critically analyzes the relative strengths and limitations of rapid and traditional transport modes, with a focused examination of the dialectical effects of HSR development, aiming to inform global policy deliberation.

In terms of speed and efficiency, HSR and AIR markedly outperform traditional modes. HSR substantially reduces intercity travel time through high-frequency, high-speed services, particularly establishing a competitive edge within the 500–900 km range [23]. Analyzing 2010 OD data for intercity rail and air passenger flows in China, Wang et al. (2017) found that railway flow connections exhibit spatial proximity, whereas air travel flows are primarily influenced by city size and functional attributes, highlighting the efficiency advantage of rapid transport over long distances [19]. In contrast, traditional road and conventional rail are limited in speed and more constrained by geographical space. Comparing multi-modal flows (long-distance bus, HSR, and flights), Wang et al. (2019) noted that road transport is constrained by provincial administrative boundaries, HSR connections exhibit corridor effects, and air transport reflects high-level socioeconomic linkages at national and regional scales; traditional modes like long-distance buses see their markets significantly affected by HSR, with pronounced distance-decay effects [20]. This efficiency differential manifests economically as rapid transport more readily channeling production factors toward hub nodes, whereas traditional modes may sustain a more dispersed distribution pattern [56]. However, this efficiency advantage is not absolute. In remote or topographically complex areas, traditional roads may offer greater inclusivity due to their flexibility and lower barriers, though as Asher and Novosad (2020) found in their study of rural roads in India, while roads facilitated labor movement out of agriculture, they did not significantly improve incomes or assets, indicating limited potential for traditional transport in fostering grassroots development [49].

Regarding economic impact, the role of HSR and AIR in regionally coordinated development remains highly contested. As outlined in Section 2.1.1, proponents highlighting HSR’s promotion of regional coordination emphasize its diffusion effects and convergence tendencies, whereas opposing views argue that HSR and AIR can intensify core-periphery divergence and regional inequality. In contrast, traditional transport modes like road and conventional rail may play a more moderate role in regional development. Analyzing transport infrastructure from a lifecycle perspective, Gao et al. (2021) found that transport investment during the construction phase may widen regional economic disparities, whereas during the operational phase, transport mileage and services exhibit coexisting narrowing and widening effects on disparities, with roads and railways having heterogeneous impacts across regions—eastern regions being more sensitive to road mileage, western regions to railway mileage [57]. In a study in Indonesia using an instrumental variable approach, Gertler et al. (2024) found that road maintenance yielded positive welfare effects by promoting formal employment and increasing incomes, with a benefit-cost ratio of 2.3, suggesting that traditional transport may offer more sustainable outcomes in grassroots development contexts [53].

In terms of accessibility and spatial structure, HSR and AIR reshape regional spatial patterns by altering time-space distances, yet this process often accompanies the strengthening of core-periphery structures. Applying accessibility methods, Wang and Ding (2011) indicated that HSR construction generates time-space convergence effects, driving the reconfiguration of production factors and thereby influencing the restructuring of urban spatial forms [21]. Using accessibility analysis and a Geographically and Temporally Weighted Regression (GTWR) model in a study of Southwest China, Zhou et al. (2024) found that HSR brought significant accessibility and socio-economic development benefits but widened the gap between HSR-connected and unconnected cities, with small and medium-sized cities along the mid-sections of HSR lines benefiting less. Such spatial differentiation is less common with traditional transport [58]. Wang et al. (2019) emphasized that road transport is noticeably constrained by geographical space, whereas air transport possesses superspatial connectivity, not adhering to the distance decay law. However, the limitation of traditional transport lies in its lower efficiency, potentially hindering effective cross-regional integration [20]. Based on data from 36 major Chinese cities, Jiang et al. (2023) compared the accessibility of HSR and aviation, finding air travel networks to be fairer than HSR networks; yet traditional road and conventional rail may provide more balanced coverage in remote areas, albeit with weaker overall impact [51].

A critical synthesis of the existing literature reveals that the comparison between rapid and traditional transport involves not only technical efficiency but also issues of equity and sustainability in regional development. The “double-edged sword” effect of HSR and AIR stems from their inherent duality: on one hand, they promote factor mobility and economic growth by reducing transaction costs and enhancing market accessibility [40,59]; on the other, they may reinforce existing economic geography, leading to “winner-take-all” phenomena. Analyzing the Guiyang-Guangzhou HSR using NPP-VIIRS remote sensing data and a PSM-DID method, Liang et al. (2020) found that HSR had a greater impact on less developed areas but did not generate significant corridor effects, indicating that the benefits of rapid transport may be unevenly distributed [60]. In contrast, traditional transport like road and conventional rail, while slower, may support grassroots development and employment through broader network coverage. Studies by Asher and Novosad (2020) and Gertler et al. (2024) demonstrate that traditional road investments hold potential for facilitating labor transfer and local welfare improvement, though their effectiveness is contingent on institutional and economic contexts [49,53].

This comparative analysis deliberately focuses on HSR and AIR networks, to the exclusion of traditional rail, road, and other transportation methods, based on a clear theoretical and methodological rationale central to our research question. Our investigation is predicated on the distinct economic geography shaped by rapid passenger transport systems, which primarily facilitate the movement of people, knowledge, and high-value services—key drivers of regional economic coordination in the modern era. While traditional rail remains indispensable for freight logistics and low-cost passenger services, its operational paradigms, underlying technologies, and consequent economic impact mechanisms (e.g., heavy-industry supply chains, bulk commodity flows) differ fundamentally from those of HSR and AIR [17,19]. By confining our scope to these two modes, we ensure analytical coherence, as HSR and AIR constitute a comparable pair: they are both capital-intensive networks characterized by high speed, compete for similar market segments of time-sensitive passengers, and have been shown to exert significant yet distinct influences on regional development through accessibility and agglomeration dynamics [15,18]. This focused approach allows for a cleaner identification of their respective comparative advantages and synergistic potential in shaping CRED, without the confounding effects of a heterogenous third mode with divergent functions. The structural comparability of HSR and AIR networks, as revealed by complex network theory [17,19], further justifies their parallel examination, enabling a consistent application of social network analysis metrics to decipher their unique roles in the spatial economy.

In summary, the impact of rapid transit networks on CRED constitutes a research domain characterized by significant tension, lacking a simple “yes” or “no” answer. Whether the net effect is positive or negative depends on a complex interplay of multiple factors: the time horizon (short-run vs. long-run), the spatial scale (national, regional, or urban agglomeration), the network structure (monocentric vs. polycentric), and local endowments (industrial base vs. human resources), among others. Future research must move beyond discussions of mere “existence” to delve deeper into these boundary conditions, with increased attention directed towards AIR networks and their synergistic effects with HSR.

### 2.2. Research on the relationship between HSR and AIR networks

Research on the relationship between HSR and AIR has evolved from an initial focus on modal competition to progressively encompassing multiple dimensions, including network interaction, long-term strategic adjustments, and impacts on social welfare, reflecting a trend toward more dynamic and systemic analysis.

#### 2.2.1. Competitive dynamics and passenger diversion.

Competition is the most direct manifestation of their relationship, with extensive literature focusing on passenger diversion effects and critical distance thresholds. Large-scale empirical studies from China have provided robust global evidence. Using panel data from 138 competing routes (2007–2013), Yang et al. (2018) found that HSR entry reduced airline demand by an average of 27%, with this negative impact intensifying over the duration of HSR operation [61]. Travel time and frequency are key factors influencing air passenger volume, while fare—due to government regulation—has a relatively weaker effect. A binary logit model analysis of the Wuhan–Guangzhou corridor by Zhang et al. (2012) indicated that intense competition between HSR and AIR occurs when airfare discounts fall below 30% [62]. Estimates of the critical distance vary across studies depending on methodology and context but generally fall within the 600–1000 km range. Using a transport market share model, Ding et al. (2013) identified 500–900 km as the salient competitive range, with 692 km as the market boundary distance [23]. Sun et al. (2017) placed the market threshold at 633 km [56]. Luo et al. (2019), employing a two-stage game model, further refined the most intense competition interval to 650–850 km [63]. Meng et al. (2022) applied a passenger value-of-time theory to develop a marginal time-saving cost model. Their simulation analysis suggested that the effective competitive distance between HSR and AIR is bounded around 800 km and varies with factors such as hub connection time and operating speed, providing a theoretical explanation for the dynamic nature of the critical distance [18].

However, competitive effects are not uniformly distributed and exhibit significant spatial and route heterogeneity. Studies generally find that HSR has a greater impact on airports in small and intermediate cities than on those in large or endpoint cities [20,23]. Sun et al. (2017) further summarized general patterns of HSR’s impact on air routes, noting that the effect of a one-time, full line opening is greater than phased openings, and that routes in commercially vibrant, tourist-rich, or topographically constrained areas exhibit greater resilience [56]. Jiang and Zhang’s (2016) theoretical model revealed deeper long-term competitive implications: faced with HSR competition on trunk routes, airlines tend to adjust their network structures, increasing coverage in peripheral (regional or international) markets and evolving toward hub-and-spoke systems. Such strategic adjustments may align their networks more closely with the social optimum, thereby generating new welfare gains [64]. Wang et al. (2020), studying the spatial evolution of HSR–AIR competition networks, observed an east-to-west expansion with agglomeration characteristics, and a competitive threshold distance of 1000–1100 km influenced by factors like city size, thereby enriching the understanding of competition heterogeneity from a network perspective [14].

#### 2.2.2. Integration, environmental and social welfare.

As research has deepened, scholars have moved beyond a simplistic zero-sum game perspective to explore possibilities for integration and its broader implications. Dobruszkes and Givoni (2013) critically discussed air-rail integration, noting that while HSR with high load factors is more environmentally friendly than AIR with low load factors from both operational and lifecycle perspectives, the environmental benefits of integration may become minimal or even negative if the freed airport runway capacity is reallocated to longer-haul flights [65]. Thus, integration appears more as a commercial opportunity than an inherently sustainable option. A subsequent analysis of 161 European routes by Dobruszkes et al. (2014) confirmed that shorter HSR journey times (within approximately 2.0–2.5 hours) lead to reductions in air service, while the effect of HSR frequency is more limited, providing empirical boundaries for the necessity of integration [66].

At the social welfare level, theoretical models reveal a more complex picture. Modeling by Yang and Zhang (2012) suggested that welfare may be higher with an HSR system, particularly when airlines can engage in price discrimination (between business and leisure travelers) and the difference in travel revenue between these passenger types substantially outweighs their difference in value of time [59]. A game-theoretic model by Tsunoda (2018) provided a theoretical basis for government regulatory policy, indicating that unless the consumer benefit from HSR is sufficiently large or sufficiently small compared to AIR, “partial public regulation” emerges as a subgame perfect equilibrium. In this scenario, the HSR operator’s objective function becomes a weighted sum of profit and social welfare, offering a theoretical explanation for the joint public and private investment observed in HSR networks in Europe and Asia [67]. Numerical simulations by Yang and Zhang (2012) further analyzed welfare changes under different pricing strategies, deepening the welfare analysis [59].

However, the environmental consequences of competition may be less optimistic. A duopoly model established by D’Alfonso et al. (2015) introduced a key insight: the introduction of HSR could have a net negative environmental impact due to the traffic generation effect—the new travel demand induced by lower generalized travel costs [68]. Their analysis further indicated that when environmental externalities are incorporated into the social welfare calculus, the total social surplus under competition between the two modes could be lower than in a market served solely by AIR. This suggests that promoting HSR based solely on market competition grounds may lead to unintended negative environmental consequences.

#### 2.2.3. Network-level interactions and comparisons.

In recent years, complex network theory has been widely applied to analyze the network structures and interactions of HSR and AIR. Based on 2019 timetable data, Cao and Du (2022) constructed undirected network models and found the HSR network to be more compact with better connectivity, whereas the AIR network nodes exhibited stronger intermediacy and global accessibility [17]. A comparative study by Wang and Jing (2017) using 2010 OD data revealed that railway flow connections demonstrate spatial proximity, while air flow connections are primarily influenced by city size and functional attributes—structural differences that determine their distinct functional roles [19]. Yu et al. (2023) applied complex network theory to construct a medium- and long-distance rapid passenger transport network, analyzing its topological characteristics and robustness. They found the network possessed strong accessibility with small-world effects, and its robustness increased over time with higher resilience against random attacks, providing new metrics for network performance comparison [69]. Wang et al.‘s (2019) comparison of spatial cascade systems from a multi-modal flow perspective further clarified the suitable spatial scales and administrative scopes for each transport mode, as well as the constraining role of geographical space [20].

The latest research has begun focusing on integrated networks. A complex network analysis of China’s air-rail integrated network by Lu et al. (2025) found that it not only exhibits small-world properties but also demonstrates broad-scale characteristics [70]. Through hybrid modularization, the integrated network combines the centralized structure of the AIR network with the corridor-based design of the HSR network, achieving higher efficiency. However, this integration also creates reliance on high-centrality nodes, potentially introducing new resilience challenges. This suggests that their future relationship will evolve from “competition” to “coopetition,” with synergistic models like air-rail intermodality becoming an important research direction. Su et al. (2025), constructing a competition network based on train and flight schedules, found competition intensity follows an “inverted U-shape,” peaking in the 700–1300 km range. Furthermore, this competition itself promotes urban economic growth, particularly benefiting cities with stronger economic foundations, proposing a novel “competition-promotes-growth” perspective [71].

Passenger behavior research forms the foundation for understanding this coopetitive relationship. Cheng et al. (2021), employing stated preference surveys and a mixed logit model, identified access/egress time and in-vehicle time as primary factors, with the ML model better capturing the heterogeneity in passenger choice behavior [72]. Lin (2018) compared the competitive advantages of AIR and HSR from a customer perceived value perspective and proposed countermeasures for the aviation industry [73]. International research, spanning González-Savignat’s (2004) analysis of the Madrid-Barcelona corridor, Park and Ha’s (2005) study of Korea’s KTX, Behrens and Pels’s (2012) work on the London-Paris market, Mancuso’s (2014) investigation of the Milan-Rome link, and Terpstra and Lijesen’s (2015) research on airport competition, has consistently validated the critical role of travel time, frequency, and cost in modal competition, thereby enriching the international reference frame for China-specific studies [74–78].

In summary, the relationship between HSR and AIR constitutes a multi-tiered, dynamically evolving complex system. Research has progressed from merely demonstrating the existence of competition to dissecting its conditions and heterogeneous impacts and further expanded to encompass comprehensive assessments of long-term strategic adjustments, network integration, and environmental and social welfare. This evolution provides a rich theoretical foundation for optimizing integrated transport systems.

### 2.3. Differences in the impact of HSR and AIR on coordinated regional economic development (CRED)

Owing to their systematic differences in intrinsic techno-economic characteristics and network structures, HSR and AIR exhibit distinct yet complementary impacts on CRED in terms of spatial orientation, operational scales, and underlying mechanisms. This functional differentiation lays a theoretical groundwork for achieving synergistic effects through air-rail cooperation.

#### 2.3.1. Functional differentiation in transport characteristics and network structures.

The fundamental distinctions between HSR and AIR stem from their inherent transport characteristics. AIR holds an absolute advantage in speed, granting it core competitiveness for long-distance travel—a trait particularly suited to China’s vast territory and the considerable distance between its eastern and western regions [56]. In contrast, HSR holds a comparative advantage in short-to-medium distance transport, with large capacity and high frequency, enabling it to support high-intensity intercity connections [15]. While focusing on imbalances, the study by Yang and Zhang (2023) on regional integration in the Yangtze River Delta reflects the critical role of high-speed transport networks, whether HSR or AIR, in connecting developed economic zones [2].

These characteristic differences directly shape their distinct network structures. Numerous studies applying complex network theory consistently show that AIR networks exhibit more “superspatial” properties. Jiang et al. (2023) found air travel networks to be fairer than HSR networks across all measured accessibility dimensions [51]. Wang et al. (2019) noted that, due to its superspatial connectivity, air transport does not adhere to the distance decay law and shows less pronounced community structure [15]. Its network nodes (airports) exhibit stronger intermediacy and global accessibility, effectively linking eastern and western regions, as well as core and peripheral areas [17,19]. Consequently, the AIR network holds irreplaceable strategic value in promoting interregional coordination, particularly in connecting the developed eastern and less-developed western parts of China. The empirical study by Zuo and Chen (2025) supports this, finding that airports effectively reduce county-level inequality, especially in non-central cities and areas with high unemployment [79]. The theoretical LS model constructed by Xu and Cheng (2021) elucidates the mechanism: the westward opening under the “Belt and Road” Initiative can narrow the east-west economic gap by reducing transport costs and reshaping the economic geography of trade and industry. This indirectly underscores the potential strategic role of the AIR network in facilitating westward opening and promoting interregional coordination [80].

In contrast, the HSR network demonstrates strong “geographical constraints” and “corridor effects.” Its network is denser, more compact, and offers better nodal connectivity, yet it is significantly influenced by geographical distance and administrative systems, with expansion showing proximity and regional characteristics [15]. Passenger flows are highly concentrated within and between eastern, and central regions [17]. Therefore, HSR is more adept at strengthening intra-regional economic linkages, particularly suited to fostering synergistic development within urban agglomerations and between central cities and their hinterlands—for instance, guiding the formation of industrial “golden corridors” along transport axes [21]. A study of Southwest China by Zhou et al. (2024), using a GTWR model, precisely revealed the spatially differentiated impacts of HSR: while it brings significant accessibility and socio-economic benefits, it also widens the gap between connected and unconnected cities and exerts relatively weaker effects on small and medium-sized cities along the line. This vividly illustrates the limitations of the HSR “corridor effect” and its potential to create new imbalances in developing regions [58].

#### 2.3.2. Complementarity in spatial effects and coordination scales.

Based on these differences, the two modes naturally assume distinct yet complementary roles in terms of spatial focus and scale when promoting CRED. AIR’s “point-based” development pattern, relying on airports and flexible route networks, enables it to reach vast, sparsely populated, and less developed regions—such as Western and Northeastern China—with relatively low cost. These areas often lack the passenger demand necessary to support HSR services [15]. Consequently, AIR primarily operates at the “macro” scale, facilitating coordinated development across the nation or between major regions like the eastern and western parts of the country. By connecting core and peripheral areas, it helps integrate remote regions into the national unified market. A study of the Guiyang-Guangzhou HSR by Liang et al. (2020) found that HSR had a greater impact on less developed areas and those farther from central cities, primarily through “investment effects” and “industrial structure effects,” yet it did not generate significant corridor effects. This suggests that in extremely underdeveloped regions, AIR may still serve as an indispensable initial catalyst [68].

In contrast, HSR’s “corridor-based” development concentrates its influence along specific lines. It plays a more substantial role at the “meso” scale—within urban agglomerations and regions—by extending the reach of central cities, fostering metropolitan integration, and strengthening urban-rural linkages [21]. The study on polycentric structures by Xu (2023) essentially supports HSR’s potential positive role at this scale [36]. However, as noted previously, this effect is conditional; if mismanaged, it can reinforce core-periphery structures and inhibit coordinated development [33]. Jia et al. (2017), analyzing from the perspective of heterogeneous space and matching effects, demonstrated that the economic impact of HSR varies by line and local endowment. They argue that cities must adjust their local factor endowments to adapt to HSR; otherwise, they risk being marginalized. This insight underscores the demanding preconditions for HSR to genuinely foster regional coordination [47].

Compared to the extensive research on HSR, studies examining the impact of AIR networks on CRED are relatively scarce, yet their findings are equally complex. Yin et al. (2022) found that civil aviation exerts a significant positive influence on the agglomeration and radiation effects of national central cities, with larger hub airports playing a more substantial role [24]. A longitudinal analysis using nighttime light data by Zuo and Chen (2025) revealed that airport construction, on average, reduces county-level inequality by 0.009 units. This effect is more pronounced in non-central cities and regions with higher unemployment rates, operating through mechanisms such as fostering entrepreneurship, increasing tertiary sector employment, and attracting foreign investment [79]. However, research by Mao and Chen (2023) highlights the conditional nature of this effect. They found that the disparity-narrowing impact of airport construction via increased investment only emerged after the 2002 airport localization reform, was significant for regional airports, but was not observed for hub and trunkline airports [55]. Wang et al.‘s (2006) early study on the spatial pattern of China’s airport system revealed its uneven distribution and a “tripod-shaped” spatial structure, providing a foundational understanding of the historical context shaping the current effects of the AIR network [22]. Hao (2022) contributed from a freight perspective, developing a theoretical framework to analyze the impact of air cargo networks on agglomeration economies and regional economies under the competitive pressure of HSR. The study found significant regional and hierarchical heterogeneity in these effects, thereby enriching the dimensionality of research on AIR’s economic impact [81].

#### 2.3.3. Exploring pathways from competition to synergy.

Recognizing the distinct advantages of HSR and AIR, scholars have begun exploring pathways through which their cooperation—specifically “air-rail intermodality”—could potentially promote CRED. The core of such cooperation lies in functional integration and network coupling. Theoretically, collaboration enables a perfect complementarity between speed and coverage: AIR assumes responsibility for long-distance, high-speed trunk transport between city pairs, while HSR efficiently extends air service accessibility to broader hinterlands via ground networks, together forming a seamlessly connected integrated transport system. Research by Sun et al. (2024) indicates that transport networks enhance the sustainability of urban agglomeration spatial structures by accelerating regional hierarchies and connectivity, while generating positive spatial spillovers—providing empirical support for the notion that air-rail cooperation can improve overall network performance [16].

This integration can generate synergistic effects. First, it expands market reach. Through air-rail intermodality, the effective hinterland of an airline hub can extend hundreds of kilometers, significantly enhancing the hub airport’s passenger catchment capacity while enabling residents of more second- and third-tier cities to conveniently access international air services [64]. Second, it contributes to optimizing resource allocation. Cooperation can incentivize airlines to concentrate resources on long-haul routes where they hold competitive advantages, while ceding short- and medium-haul markets to the more efficient HSR, thereby improving the overall efficiency of the transport system [65]. Finally, from a CRED perspective, air-rail cooperation can more precisely serve the development needs of different region types. It can maintain and strengthen rapid connections between strategic national regions via the AIR network, while simultaneously deepening intra-regional integration and interaction through the HSR network, thus exerting influence at both macro and meso scales to jointly promote coordinated regional development. Wang and Kong (2021) studied the spatial spillover effects of airport economies on regional economic growth from a “dual circulation” perspective, finding significantly positive and increasing effects—providing theoretical support for the “airport-industry-city” development model anchored around hub airports within air-rail cooperation [82]. The unified “agglomeration-network externality” framework proposed by Fan and Zang (2026) systematically explains how HSR networks promote intercity collaborative innovation through three mechanisms: efficiency enhancement, global spillover, and synergy amplification. This analytical framework can be effectively extended to understand the considerable potential of air-rail cooperation in facilitating regional knowledge flows and innovation synergy [83].

However, realizing air-rail cooperation faces numerous challenges, including seamless physical connectivity, integrated ticketing systems, coordinated schedules, and mechanisms for benefit sharing [66]. Although the integrated network study by Lu et al. (2025) demonstrated potential efficiency gains, it also warned of the concomitant increase in systemic risks [70]. Therefore, future research and practice must delve deeper into specific models of air-rail cooperation, governance mechanisms, and the quantified impact on CRED, thereby translating theoretical complementarity into tangible developmental momentum. Research by Wei et al. (2024) on the comprehensive impact of HSR and AIR on tourism development found that AIR operation and the concurrent operation of air and rail had significant positive effects, whereas HSR operation alone showed unstable impacts. This provides direct empirical motivation for air-rail cooperation in promoting regional development within specific sectors like tourism [84].

In summary, HSR and AIR are not mutually exclusive in promoting CRED; rather, they possess distinct characteristics and complementary functions. The future development of China’s comprehensive transport system should transcend simplistic debates over which mode is superior and instead focus on how scientific top-level design and institutional innovation can guide them from competition toward synergy—collectively forming a modern, highly efficient rapid transport backbone that supports the national strategy for coordinated regional development.

### 2.4. Differences and contributions

Based on a systematic review and critical analysis of the existing literature, this research field, while yielding substantial findings, still exhibits several notable research gaps and dimensions warranting further exploration. These gaps constitute the starting point and potential contributions of this paper.

First, regarding research conclusions, a fundamental divergence persists concerning the net effect of rapid transit networks on coordinated regional economic development. Numerous studies confirm their diffusion effects and convergence role by enhancing accessibility, facilitating factor mobility, and optimizing spatial structure [25, 26]. However, equally robust opposing evidence suggests they may exacerbate regional disparities by reinforcing agglomeration and inducing polarization [28,29]. This divergence highlights the complexity and context-dependent nature of the underlying mechanisms, indicating that merely demonstrating the “existence” of effects is insufficient. Future research must strive to uncover the boundary conditions that yield these divergent outcomes, such as the stage of development, regional spatial structure, and local industrial and institutional endowments [31,36].

Second, there is a noticeable disproportion in research focus, characterized by a heavy emphasis on HSR and a relative neglect of AIR, with investigations into their synergistic effects still in their infancy. The vast majority of existing literature concentrates on the economic impacts of HSR, while dedicated, systematic studies on the AIR network remain relatively scarce [24,79]. Although the competitive relationship between HSR and AIR has been thoroughly discussed [61,63], research that treats them as an integrated transport system to deeply investigate cooperative models and their synergistic mechanisms for promoting regional coordination is notably underdeveloped [65,70]. This research gap urgently needs addressing, particularly within China’s strategic context of building a modern comprehensive transport system.

Third, concerning the measurement of transport networks, existing studies often rely on binary variables indicating “infrastructure presence,” while in-depth characterization of “network structure and function” requires strengthening. Most empirical studies use variables such as “whether HSR is operational” or “whether an airport exists” as core explanatory variables [35,55]. While intuitive, this approach fails to capture the economic geography implications embedded in deeper features like network topology, connection strength, and nodal centrality. Although pioneering work has been done using complex network theory [17,32], systematically integrating refined network structural indicators into empirical models examining the impact mechanisms on coordinated development remains a key direction for future research.

Fourth, regarding the scale and dimensions of analysis, existing research often consists of single-scale case analyses, lacking multi-scale, multi-dimensional systematic comparison and integration. Numerous studies are conducted at the city, urban agglomeration, or provincial level, but few systematically compare the differential impacts of rapid transit networks on regional coordination across different spatial scales within a unified framework [33]. Simultaneously, measurements of coordinated development predominantly focus on the economic dimension, while relative insufficient attention is paid to social dimensions such as innovation, income, and public services [9,10], failing to fully reflect the rich connotations of coordinated development.

Finally, in terms of theoretical perspectives and methods, although some studies touch upon theories like New Economic Geography and network science, efforts to construct a unified analytical framework integrating “transport characteristics - network structure - spatial effects” are still lacking. While the functional differentiation and spatial division of labor arising from the distinct techno-economic characteristics of HSR and AIR have been described to some extent [15], a middle-range theoretical framework that succinctly summarize their differentiated pathways of impact and ultimately points to possibilities for synergy is absent. Methodologically, although quasi-natural experiments are widely employed, addressing treatment effect heterogeneity, accurately measuring spatial spillovers, and capturing long-term dynamic effects remain ongoing challenges [47,58].

In summary, the contribution of this study lies in addressing the aforementioned research gaps by simultaneously focusing on both HSR and AIR networks and preliminarily exploring their cooperative potential; by introducing a network structure perspective to move beyond the simplistic “connection/no connection” logic; and by employing multi-scale comparative analysis to reveal the hierarchical differences in their impacts. Ultimately, it aims to provide a more comprehensive, multi-dimensional, and in-depth analysis for understanding the complex role of rapid transit networks in promoting China’s coordinated regional development.

## 3. Theoretical framework

Based on the original intention of China’s strategies to promote coordinated regional economic development (CRED) and the series of policies implemented, the main purpose of promoting CRED is to solve the problems of unbalanced and uncoordinated regional economic development, differentiated development trends, and polarized development dynamics [85]. The core objective of this study is to reveal the comparative advantages of HSR and AIR networks in promoting CRED across different spatial scales. To achieve this, we move beyond the traditional approach of analyzing individual regions and instead adopt an inter-regional “regional pair” perspective. This analytical framework is grounded in the theories of New Economic Geography and network economics, which posit that economic interactions and development disparities are inherently relational and shaped by the connections between regions. Therefore, the research on CRED must be conducted from the perspective of comparative analysis between regions.

CRED is fundamentally manifested in the economic linkages, development gaps, and growth differentials between regions. Therefore, measuring CRED and its transportation drivers requires a dyadic analysis of “regional pairs.” Furthermore, the impacts of transportation networks are not monolithic; they are hypothesized to vary significantly depending on the spatial scale of interaction. An AIR network might be most effective in bridging vast distances between macro-regions, while HSR might excel in integrating economies within a provincial or urban agglomeration scale.

To cleanly identify these heterogeneous effects and test for the potential synergy between HSR and AIR, it is methodologically crucial to construct separate network samples at distinct spatial dimensions. This design allows us to isolate the effect of, for instance, the macro-regional AIR network from the provincial or city-level networks, thereby preventing confounding influences and enabling a clear comparison of their comparative advantages.

**Fig 2:**
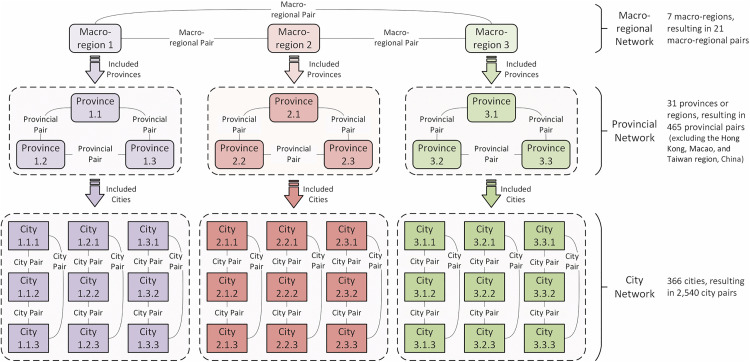
The separation methods for the macro-regional, provincial, and city networks.

Consequently, as illustrated in Fig 2, we construct our analysis around three distinct types of “regional pairs”. The research subjects selected in this study are “regional pairs” consisting of two regions. Meanwhile, in order to completely separate the effects of the HSR network and the AIR network at the macro-regional, provincial, and urban dimensions, and to extract and compare their effects at each dimension, this study will construct network samples at three dimensions to achieve the research objectives. As shown in Fig 2, the construction of the network samples at the three dimensions is as follows:

(1) At the macro-regional dimension, China is divided into 7 major regions according to the jurisdiction of the regional air traffic control bureaus of the Civil Aviation Administration of China. Pairing these 7 regions without considering the direction of flow results in 21 “macro-regional pairs”, which are used to capture large-scale, cross-regional coordination.
(2) At the provincial dimension, based on China’s provincial administrative divisions, excluding the Hong Kong, Macao, and Taiwan region, there are 31 regions. Pairing the different provinces within the same macro-region without considering the direction of flow results in 465 “provincial pairs”, which are used for coordinating analysis at the provincial-intermediate level.
(3) At the city dimension, based on China’s prefecture-level administrative divisions, excluding Hong Kong, Macau, and Taiwan, and including the province-level cities, there are 366 cities. Pairing the different cities within the same province without considering the direction of flow results in 2,540 “city pairs”, which are used to explore fine-grained coordination within provinces.

This multi-scalar “regional pair” framework provides the theoretical and empirical foundation for the model specification and variable construction that follows in the Methodology section.

## 4. Model and variables

### 4.1. Sample selection

Guided by the theoretical framework established in Section 3, we construct network samples at three spatial dimensions. In terms of the sample years, China began to implement the “*Measures for the Management of Subsidies for Civil Aviation Small and Medium-sized Airports*” and the “*Measures for the Management of Subsidies for Regional Aviation*” in 2008 to promote the development of regional air transportation, which has a positive impact on driving the development of air transportation in relatively underdeveloped regions. The latest “*China Civil Aviation Statistical Yearbook (2024)*” has published the national civil aviation route operation data for 2023. Considering these two factors, this study selects the historical sample period from 2008 to 2023. Concurrently, given the substantial impact of external containment measures on the transport sector during the COVID-19 pandemic, the 2020–2022 samples were excluded from this study. Meanwhile, to examine the latest situation of regional economic coordination and the development of HSR and AIR networks, this study selects September 2023 as the current sample period in Section 4.

### 4.2. Variables

#### 4.2.1. Coordinated regional economic development (CRED).

Regional economic coordinated development reflects both the convergence in economic outcomes and the intensity of interregional interactions [31]. We measure CRED through three complementary dimensions: the gap in per capita GDP levels (capturing static disparity), the strength of economic connections (reflecting dynamic integration), and the difference in economic growth rates (indicating trend convergence). The construction of the CRED index proceeds in two steps. First, we calculate three component indicators for each regional pair (*p*, *q*) in year *t*. Economic Development Gap (*Ed*) measured as the absolute difference in per capita GDP between two regions, serving as a negative indicator of coordination, as shown in Equation (1). Economic Connection (*Er*) captured through a gravity-model inspired measure of economic interaction intensity, serving as a positive indicator, as shown in Equation (2). Economic Growth Difference (*Ef*) represented by the absolute difference in GDP growth rates, serving as a negative indicator, as shown in Equation (3).


$$Ed_{pq}^{t}=\left|\frac{pgdp_{p}^{t}-pgdp_{q}^{t}}{Max\left(pgdp^{t}\right)-Min\left(pgdp^{t}\right)}\right|$$ (1)

$$Er_{pq}^{t}=\frac{\sqrt{gdp_{p}^{t}pop_{p}^{t}}\times \sqrt{gdp_{q}^{t}pop_{q}^{t}}}{dis_{pq}^{2}}$$ (2)

$$Ef_{pq}^{t}=\left|gdpth_{p}^{t}-gdpth_{q}^{t}\right|$$ (3)

$$CRED_{pq}^{t}=w_{pq}^{1t}Ed_{pq}^{t}+w_{pq}^{2t}Er_{pq}^{t}+w_{pq}^{3t}Ef_{pq}^{t}\left(w_{pq}^{2t}\geq 0,w_{pq}^{1t},w_{pq}^{3t}\leq 0\right)$$ (4)

In the formulas, subscripts *p* and *q* denote two regions without distinguishing the direction of flow, and *t* represents years. The *gdp* refers to the regional gross domestic product, *pop* refers to the total resident population of the region, *dis* represents the mean spatial distance between the two regions, *pgdp* refers to the per capita regional GDP within the region, and *gdpth* represents the growth rate of the regional GDP within the region.

Then, we employ the entropy weight method to determine the optimal weights for the three indicators ($w_{pq}^{1t}$ - $w_{pq}^{3t}$) in year *t* and integrate them into the comprehensive CRED index as shown in Equation (4). The application of the entropy weight method follows the research by Huang (2008) [86].

#### 4.2.2. Evaluation indicators for the HSR network and AIR network.

1 Network Scale Measurement

The development scale of high-speed rail (HSR) and aviation (AIR) networks is quantified using service frequency, which directly reflects connection intensity and accessibility. For each regional pair, the scale is defined as the total number of direct flights (for AIR) or the total number of HSR departures (for HSR) between all city pairs within the two regions. This research denotes the undirected transport network connectivity matrix for regional pair *pq* in year *t* as $A_{pq}^{t}$. An element $a_{pqij}^{t,hsr}$ in matrix $A_{pq}^{t,hsr}$ represents the number of HSR departures between city *i* in region *p* and city *j* in region *q* in year *t*, while an element $a_{pqij}^{t,air}$ in matrix $A_{pq}^{t,air}$ denotes the corresponding total number of flights. Consequently, $Scale_{pq}^{t}$ is calculated as shown in Equation (5) below, where $Scale_{pq}^{t,air}$ corresponds to the AIR network scale and $Scale_{pq}^{t,hsr}$ corresponds to the HSR network scale.


$$\begin{array}{c} \begin{array}{c} A_{pq}^{t}=\left(\begin{array}{ccc} {}*20ca_{pq11}^{t} & \cdots & a_{pqn1}^{t}\\ \vdots & \ddots & \vdots \\ a_{pq1n}^{t} & \cdots & a_{pqnn}^{t} \end{array}\right)\\ Scale_{pq}^{t}=\sum_{i=1}^{n}\sum_{j=1}^{n}a_{pqij}^{t} \end{array} \end{array}$$ (5)

2 Network Structure Quality

Based on the theoretical analysis results and referencing relevant research, this study introduces the following indicators from social network analysis to measure the development level of the transportation networks: average degree (*AveDegree*), average path length (*AveDistance*), path length deviation (*SDDistance*), and average betweenness centrality (*AveBetween*). Among them, average degree and average betweenness centrality are used to measure the centrality of the transportation network, average path length is used to measure the connectivity of the transportation network, and path length deviation is used to measure the balance of the transportation network.

Degree centrality measures the extent to which a node in a network is directly connected to all other nodes. In a transportation network, degree centrality refers to the number of node cities that are directly connected to a given node city, which is mainly used to measure the importance and influence of that node city in the entire network. In a transportation network, the higher the degree centrality of a node city, the more cities it is directly connected to and the more extensive the direct transportation connections of that city with other cities. This means that the city is more centrally located in the transportation network and has greater “power” in the network, indicating its higher importance in the network. This study defines the average degree (*AveDegree*) of the transportation network as the average of the degree centralities of all nodes in the network, representing the “direct” connection density between cities within the network. The expressions for degree centrality and average degree are:


$$\begin{array}{c} Degree_{pqi}^{t}=\sum_{j=1}^{n}a_{pqij}^{t}\\ AveDegree_{pq}^{t}=\frac{\text{1}}{n}\sum_{i=1}^{n}\sum_{j=1}^{n}a_{pqij}^{t} \end{array}$$ (6)

Where *Degree* and *AveDegree* represent degree centrality and average degree, respectively. *n* represents the number of nodes in the network, and the other symbols are the same as in Equation (5).

Path length (*Distance*) measures the connection efficiency between two nodes in the network. In a transportation network, the “shortest path” refers to the path with the fewest transportation lines connecting two transportation node cities, and the path length is the number of transportation lines on this “shortest path” (defined as $d_{ij}$). In a transportation network, the smaller the path length between two node cities, the higher the efficiency of the connection between that “city pair” and the greater the contribution of that “city pair” to the efficiency of the transportation network. The average path length (*AveDistance*), also known as the characteristic path length or average distance of the network, is the average of the path lengths between any two nodes in the network, which in a transportation network indicates the overall efficiency of transportation connections between node cities. This study further defines the path length deviation (*SDDistance*) as the standard deviation of the path lengths between any two nodes in the network, which in a transportation network indicates the degree of difference in the efficiency of transportation connections between node cities. The expressions for path length, average path length, and path length deviation are:


$$\begin{array}{c} Distance_{pqij}^{t}=d_{pqij}^{t}\\ AveDistance_{pq}^{t}=\frac{\text{1}}{\frac{\text{1}}{\text{2}}n(n-1)}\sum_{i\geq j}^{n}d_{pqij}^{t}\\ SDDistance_{pq}^{t}=\sqrt{\frac{\text{1}}{n}\sum_{i\geq j}^{n}{\left(d_{pqij}^{t}-AveDistance_{pq}^{t}\right)}^{2}} \end{array}$$ (7)

Where *Distance*, *AveDistance*, and *SDDistance* represent path length, average path length, and path length deviation, respectively, and $d_{pqij}^{t}$ represents the number of edges in the shortest path from node *i* to node *j*, and the other symbols in the formula have the same meaning as in Equation (5).

Betweenness centrality reflects the degree of control an actor has over resources. If a node is on the shortest path between many other node pairs, we say that node has a higher betweenness centrality, as it acts as a bridge connecting different actors. If a node’s betweenness centrality is 0, it means the node cannot control any other actors and is on the periphery of the network. The higher the betweenness centrality, the stronger the ability to control others. In a transportation network, if a node city is on the shortest path between many other node cities, its betweenness centrality is considered higher, indicating its ability to control the transportation connections between other cities, reflecting the role of the city as an “intermediary” and “bridge”. Average betweenness (*AveBetween*) is the average betweenness centrality of all nodes in the network, which represents the degree of the network’s clustering around core nodes. If the overall network has a higher average betweenness, it means there is a “center” in the network, and a hierarchical “subordinate” relationship is formed between the core nodes and non-core nodes, which promotes the formation of strong relationships between them and enhances the overall network’s connection strength and execution [87]. Therefore, this study defines the average betweenness (*AveBetween*) of the transportation network as the average of the betweenness centralities of all nodes in the network, representing the degree of clustering around the core nodes in the transportation network. The expressions for node betweenness centrality and network average betweenness are:


$$\begin{array}{c} Between_{pqi}^{t}=\sum_{j>k}^{n}\frac{d_{pqjk}^{t}\left(i\right)}{d_{pqjk}^{t}}\\ AveBetween_{pq}^{t}=\frac{\text{1}}{n}\sum_{j>k}^{n}\frac{d_{pqjk}^{t}\left(i\right)}{d_{pqjk}^{t}} \end{array}$$ (8)

Where *Between* and *AveBetween* represent node betweenness centrality and network average betweenness, respectively, and $d_{pqjk}^{t}\left(i\right)$ represents the number of shortest paths from node *j* to node *k* that pass-through node *i*, and the other symbols in the formula have the same meaning as in Equation (5).

Since this study focuses on the “direct” connection effect, “intermediary” connection effect, connection efficiency, and difference of the HSR and AIR networks, it selects the average degree centrality, average betweenness centrality, average path length, and path length deviation of the HSR and AIR networks to represent the “direct” connections, “intermediary” connections, and connection efficiency and difference between cities, respectively, to analyze the quality of the HSR and AIR network structures. This study uses the entropy weight method to calculate the weights of each indicator, and the comprehensive index expression for the quality of the HSR and AIR network structures is as follows:


$$\begin{array}{c} Net_{pq}^{t}=w_{pq}^{1t}AveDegree_{pq}^{t}+w_{pq}^{2t}AveBetween_{pq}^{t}+w_{pq}^{3t}AveDistance_{pq}^{t}+w_{pq}^{4t}SDDistance_{pq}^{t}\\ \left(w_{pq}^{1t},w_{pq}^{2t}\geq 0,\;w_{pq}^{3t},w_{pq}^{4t}\leq 0\right) \end{array}$$ (9)

Where $Net_{pq}^{t}$ represents the comprehensive index of the HSR or AIR network structure quality within the regional pair *pq*, $Net_{pq}^{t,air}$ represents the quality of the air route network structure, and $Net_{pq}^{t,hsr}$ represents the quality of the HSR network structure. $w_{pq}^{1t}$ - $w_{pq}^{4t}$ are the weights of the four indicators calculated annually using the entropy weight method, where $AveDegree_{pq}^{t}$ and $AveBetween_{pq}^{t}$ are positive indicators, and $AveDistance_{pq}^{t}$ and $SDDistance_{pq}^{t}$ are negative indicators.

1. Comprehensive Development and Collaborative Effect of the HSR Network and AIR NetworkThe development of transportation networks is mainly reflected in two aspects: transportation network scale and complex network structure [32]. Therefore, by incorporating the scale evaluation indicator ($Scale_{pq}^{t}$) and the structure quality evaluation indicator ($Net_{pq}^{t}$) of the HSR network and the AIR network mentioned above, this study combines the two evaluation indicators to obtain the variables representing the development level of the HSR network ($HSR_{pq}^{t}$) and the development level of the AIR network ($AIR_{pq}^{t}$), with the calculation methods as follows:

$$\begin{array}{c} HSR_{pq}^{t}=\frac{\text{1}}{\text{2}}\left(Scale_{pq}^{t,hsr}+Net_{pq}^{t,hsr}\right)\\ AIR_{pq}^{t}=\frac{\text{1}}{\text{2}}\left(Scale_{pq}^{t,air}+Net_{pq}^{t,air}\right) \end{array}$$ (10)

In addition to comparing the impacts of the HSR network and the AIR network on CRED, this study also needs to examine the impact of the collaboration between the HSR network and the AIR network on CRED. Therefore, an interaction term between the HSR network and the AIR network ($HA_{pq}^{t}$) needs to be constructed, with the calculation method as follows:


$$HA_{pq}^{t}=HSR_{pq}^{t}\times AIR_{pq}^{t}$$ (11)

#### 4.2.3. Control variables.

This study selects the following control variables based on other factors at the regional pair level that may affect the CRED level:

① Other transportation modes: highway passenger turnover (Road). Highway transportation also belongs to the transportation mode, and relevant theories can also prove that highway transportation can affect CRED. According to previous research, highway transportation can have a significant positive impact on CRED [26]. The calculation methods are the average of the highway passenger turnover of the two regions.
② Economic development characteristics: economic structure (Structure), talent resources (Edu), technical resources (Tech), labor force (Labor), and government intervention (Gov). The calculation method for regional material capital stock refers to the research of [88]. The calculation methods for the other variables are the average proportion of GDP of the tertiary industry in GDP of the two regions, the average number of college students per 10,000 people, the average number of patents granted, the average labor force of working age, and the ratio of fiscal expenditure to regional GDP of the two regions, respectively.

The variables and their symbols and calculation methods are shown in Table 1.

**Table 1:** Variable name and definition.

Variable Type	Variable Name	Variable Symbol	Calculation Method
Dependent Variable	Coordinated regional economic development	*CRED*	Equation (4)
	Gap in Regional Economic Development Level	*Ed*	Equation (1)
	Regional Economic Connections	*Er*	Equation (2)
	Regional Economic Growth Difference	*Ef*	Equation (3)
Independent Variable	HSR Network Development	*HSR*	Equation (5) to (10)
	AIR Network Development	*AIR*	Equation (5) to (10)
	Collaboration between HSR Network and AIR Network	*HA*	Equation (11)
Control Variable	Highway Passenger Turnover	*Road*	Average of highway passenger turnover of the two regions
	Economic structure	*Structure*	Average proportion of GDP of the tertiary industry in GDP of the two regions
	Talent Resources	*Edu*	Average number of college students per 10,000 people of the two regions
	Technical resources	*Tech*	Average number of patents granted in the two regions
	Labor Force	*Labor*	Average labor force of working age of the two regions
	Government Intervention	*Gov*	Average ratio of fiscal expenditure to regional GDP of the two regions

### 4.3. Model

#### 4.3.1. Baseline regression model.

This study adopts the fixed-effects ordinary least squares (OLS) regression model as the baseline regression, as shown in Model (13) to (15):


$$CRED_{pq}^{t}=\beta_{0}^{1}+\beta_{1}^{1}AIR_{pq}^{t}+\beta_{4}^{1}Control_{pq}^{t}+u_{pq}^{1}+\gamma^{t1}+\varepsilon_{pq}^{t1}$$ (12)

$$CRED_{pq}^{t}=\beta_{0}^{2}+\beta_{1}^{2}HSR_{pq}^{t}+\beta_{4}^{2}Control_{pq}^{t}+u_{pq}^{2}+\gamma^{t2}+\varepsilon_{pq}^{t2}$$ (13)

$$CRED_{pq}^{t}=\beta_{0}^{3}+\beta_{1}^{3}HA_{pq}^{t}+\beta_{2}^{3}AIR_{pq}^{t}+\beta_{3}^{3}HSR_{pq}^{t}+\beta_{4}^{3}Control_{pq}^{t}+u_{pq}^{3}+\gamma^{t3}+\varepsilon_{pq}^{t3}$$ (14)

Where *p* and *q* represent the two regions without distinguishing the direction, and *t* represents the year. $CRED_{pq}^{t}$ represents the coordinated regional economic development level, $AIR_{pq}^{t}$, $HSR_{pq}^{t}$, and $HA_{pq}^{t}$ represent the development level of the air transportation network, the development level of the HSR network, and the interaction between the air transportation network and the HSR network, respectively, between the two regions. Since $AIR_{pq}^{t}$ and $HSR_{pq}^{t}$ are both continuous variables, control for $AIR_{pq}^{t}$ and $HSR_{pq}^{t}$ needs to be included in Model (15) to avoid model specification issues. $Control_{pq}^{t}$ represents the control variables at the regional pair level, $u_{pq}$ represents the regional pair fixed effects to control for time-invariant factors at the regional pair level, $\gamma^{t}$ represents the year fixed effects to control for factors that affect all samples during the sample period, and $\varepsilon_{pq}^{t}$ is the random error term. If $\beta_{1}$ is significant and greater than 0, it indicates that the corresponding independent variable can significantly improve the CRED level.

#### 4.3.2. Dose-Response (DR) model.

In this study, the traditional ordinary least squares (OLS) model has performed an average estimation of the heterogeneous effects between regional pairs with different transportation network development levels, ignoring this difference. To better examine the heterogeneous impacts of transportation network development levels on CRED, this study adopts the DR model for the analysis. The DR model is a method for quantitatively describing the relationship between the intensity of a treatment (i.e., the “dose”) and the likelihood of an effect (i.e., the “response”) [89]. The model assumptions include: (1) the treatment is continuous; (2) individuals exhibit heterogeneous responses to observable confounding factors; (3) treatment selection may be endogenous.

Compared to traditional regression models, the DR model has the following advantages: (1) it can go beyond a single average effect by estimating the dose-response function (DRF) to provide effect estimates based on the values of the dose variable; (2) the DRF plot can provide clear and intuitive results, which is helpful for analyzing causal relationships; (3) it can study the overall distribution of causal effects, better understanding the observed pattern of effects; (4) it can incorporate counterfactual designs, enhancing the causal interpretability of the results [90].

The DR model has strong applicability in this study. The independent variables in this study include the development level of the HSR network ($HSR_{pq}^{t}$), the development level of the AIR network ($AIR_{pq}^{t}$), and the interaction term between the HSR network and the AIR network ($HA_{pq}^{t}$), all of which are continuous variables. Previous research has shown that the economic effects brought by transportation networks of different development scales exhibit heterogeneous characteristics, and the larger the scale of the transportation network, the stronger the economic effects it generates [84]. Finally, the development level of transportation networks may be influenced by CRED, as higher CRED levels indicate closer economic connections between cities and greater demand for transportation infrastructure such as HSR [32], which may lead to endogeneity in the promotion of CRED by the HSR network and the AIR network. In summary, the adoption of the DR model in this study can better capture the heterogeneous impacts of transportation network development levels on CRED, providing a new perspective for a deeper understanding of the underlying mechanisms. This study refers to the econometric models developed by Cerulli (2015) and Filippetti and Cerulli (2018) to construct the DR model, and similar model applications can also be found in the studies of Wei et al. (2024), Cerulli and Ventura (2021), Prifti et al. (2019) [84,89–92].

First, we define the continuous variable $t_{i}$, which takes values from 1 to *N*, with the range of $t_{i}$ being [0, 100], as an indicator variable for the continuous treatment variable, and $h\left(t_{i}\right)$ is a generally differentiable function of $t_{i}$. The DRF is equal to the average treatment effect (*ATE*) given the treatment level *t*, i.e., $ATE\left(t_{i}\right)$. The treatment indicator *ω* is 0 when $h\left(t_{i}\right)=0$ and 1 otherwise, corresponding to the random coefficient estimation equations without and with $h\left(t_{i}\right)$, respectively, which also correspond to the average treatment effect on the treated (*ATET*) and the average treatment effect on the non-treated (*ATENT*).

Next, given a set of *M* explanatory variables $x_{i}=\left(x_{1i},x_{2i},\ldots ,x_{Mi}\right)$, under the assumption of no confounding or conditional mean independence (CMI), both *ω* and *t* are exogenous. The estimation of the response function *y* and the setting of the parameters $h\left(t_{i}\right)$ in it can be written as Equations (15) and (16)below. Using OLS estimation, we can obtain consistent estimates of the parameters in Equation (15) and directly estimate the *ATE* from this regression.


$$E\left(y_{i}|\omega_{i},t_{i},x_{i}\right)=\alpha_{0}+\omega_{i}\cdot ATE+x_{i}\delta_{0}+\omega_{i}\cdot \left(x_{i}-\bar{x}\right)\delta +\omega_{i}\left[h\left(t_{i}\right)-\bar{h}\right]$$ (15)

$$h\left(t_{i}\right)=\lambda_{1}t_{i}+\lambda_{2}t_{i}^{2}+\lambda_{3}t_{i}^{3}$$ (16)

Where $\alpha_{0}$, $\delta_{0}$, *ATE*, *δ*, $\lambda_{1}$, $\lambda_{2}$, and $\lambda_{3}$ are the parameters to be estimated. Under the CMI assumption, using OLS to estimate Equations (15) and (16)can obtain the estimates of these parameters. These parameters can then be used to consistently estimate the *DRF*:


$$\begin{array}{c} \hat{ATE}\left(t_{i}\right)=\omega_{i}\left\{\hat{ATET}+{\hat{\lambda }}_{1}\left(t_{i}-\bar{t}\right)+{\hat{\lambda }}_{2}\left(t_{i}^{2}-\overset{―}{t^{2}}\right)+{\hat{\lambda }}_{3}\left(t_{i}^{3}-\overset{―}{t^{3}}\right)\right\}+\left(1-\omega_{i}\right)\hat{ATENT}\\ \hat{ATET}\left(t_{i}\right)=\hat{ATE}{\left(t_{i}\right)}_{t_{i}>0},\hat{ATENT}\left(t_{i}\right)=\hat{ATE}{\left(t_{i}\right)}_{t_{i}=0} \end{array}$$ (17)

Furthermore, for each dose level *t*, the confidence interval of the DR curve can be calculated. According to the research of Filippetti and Cerulli (2018) [92], the standard error of the *DRF* is as follows:


$$\begin{array}{c} {\hat{\sigma }}_{\hat{ATE}\left(t\right)}={\left(T_{1}^{2}{\hat{\sigma }}_{\lambda_{1}}^{2}+T_{2}^{2}{\hat{\sigma }}_{\lambda_{2}}^{2}+T_{3}^{2}{\hat{\sigma }}_{\lambda_{3}}^{2}+2T_{1}T_{2}{\hat{\sigma }}_{\lambda_{1},\lambda_{2}}+2T_{1}T_{3}{\hat{\sigma }}_{\lambda_{1},\lambda_{3}}+2T_{2}T_{3}{\hat{\sigma }}_{\lambda_{2},\lambda_{3}}\right)}^{1/2}\\ T_{1}=t-E\left(t\right),T_{2}=t^{2}-E\left(t^{2}\right),T_{3}=t^{3}-E\left(t^{3}\right) \end{array}$$ (18)

Finally, based on this, the confidence interval for each *t* can be obtained as shown in Equation (19). By plotting the DR curve and its confidence interval, the impact coefficient and significance of the explanatory variable *t* on CRED can be visually examined as the amount of the variable *t* changes.


$$\left\{\hat{ATE}\left(t\right)\pm Z_{\alpha /2}\times {\hat{\sigma }}_{\hat{ATE}\left(t\right)}\right\}$$ (19)

In the DR model constructed in this study, the explanatory variables are *HSRindex*, *AIRindex*, and *HAindex*, calculated as shown in Equation (20), with a range of [0, 100]. The control variables used in the estimation are consistent with the baseline OLS model, and all variables are standardized using Z-scores.


$$\begin{array}{c} HSRindex_{pq}^{t}=\frac{HSR_{pq}^{t}-\min\left(HSR\right)}{\max\left(HSR\right)-\min\left(HSR\right)}\times 100\\ AIRindex_{pq}^{t}=\frac{AIR_{pq}^{t}-\min\left(AIR\right)}{\max\left(AIR\right)-\min\left(AIR\right)}\times 100\\ HAindex_{pq}^{t}=\frac{HA_{pq}^{t}-\min\left(HA\right)}{\max\left(HA\right)-\min\left(HA\right)}\times 100 \end{array}$$ (20)

### 4.4. Data sources

(1) Transportation network data: The actual HSR train departure frequency data is currently unavailable, so this study uses the number of HSR train numbers between cities as a substitute. The railway train number is the identification code assigned by the China State Railway Group Co., Ltd. to trains of different driving directions, vehicle types, driving sections, and running times. Therefore, the number of HSR train numbers between cities can reflect the scale of HSR train departure frequency. The historical data of domestic airline flight volume is from the “*China Civil Aviation Statistical Yearbook*” from 2009 to 2024, and the historical data of HSR train numbers is from the “*National Railway Passenger Train Timetable*” from 2008 to 2016 and the 12306.cn website from 2017 to 2023. The latest status data of domestic airline flight volume is from the OAG database in September 2023, and the current status data of HSR train numbers is from the 12306.cn website crawled in September 2023.
(2) Regional economic data: The macro-economic data at the macro-regional dimension is calculated from the provincial-level data, which is obtained from the website of the National Bureau of Statistics. The macro-economic data at the prefecture-level administrative division is from the “China City Statistical Yearbook” from 2009 to 2024, with the missing regional data manually collected from the 20092024 government statistical bulletins of the respective regions, including a small number of prefecture-level administrative units and 32 provincial-level county-level administrative units. The original data required for calculating the material capital stock is from the EPS China Macroeconomic Database.

To eliminate the impact of dimensional differences between variables on the estimation results and achieve the research goal of comparing regression coefficients of different variables, all variables in this study are standardized using the Z-Score method before empirical testing.

## 5. Descriptive analysis

To preliminarily examine the coordinated regional economic development (CRED) level, the development level of the HSR network, and the AIR network in China, this study conducts a descriptive statistical analysis of the constructed variables to provide a preliminary analysis of the potential heterogeneous impacts of the HSR network and the AIR network on CRED.

### 5.1. Comparison of the scale of HSR network and the AIR network

#### 5.1.1. Temporal variation characteristics of the scale of HSR and AIR networks.

**Fig 3:**
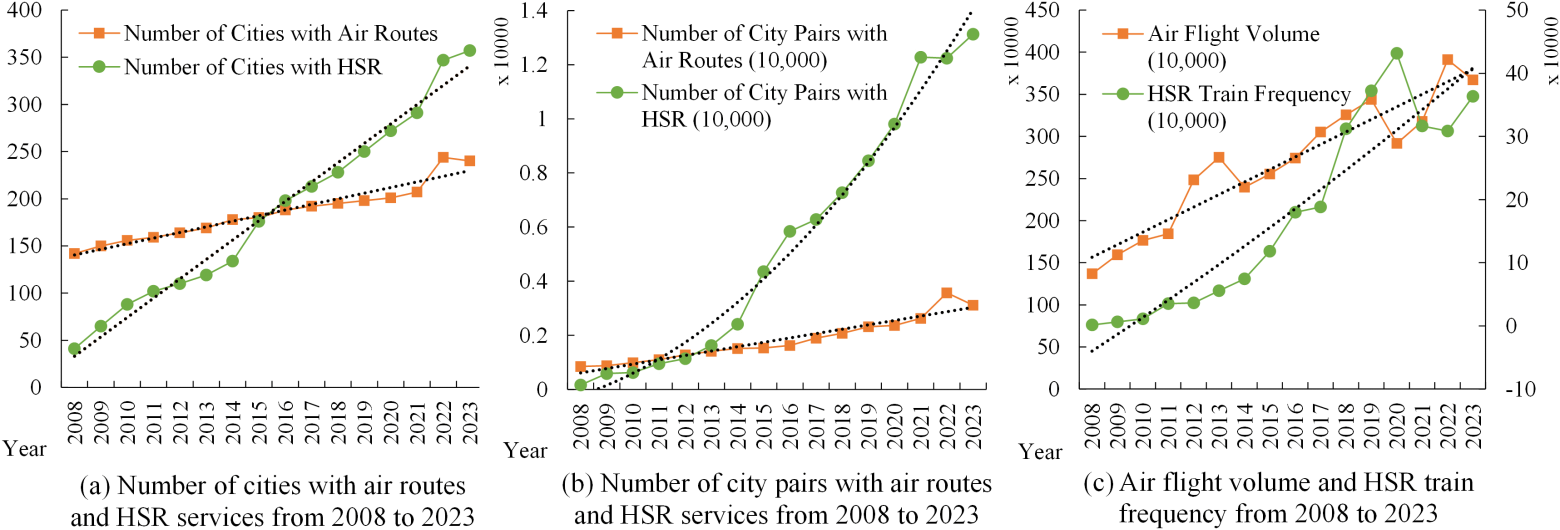
Trends of changes in the development scale of AIR and HSR networks from 2018 to 2023.

This study has constructed the AIR and HSR networks in China from 2008 to 2023, and measured the network scale from the three key elements of network nodes (transportation node cities), edges (transportation routes), and edge weights (transportation frequencies or train numbers), using linear and quadratic polynomial functions for trend fitting, as shown in Fig 3. Overall, both the AIR and HSR networks in China have been continuously expanding during 20082023, with the AIR network exhibiting a linear growth trend and the HSR network mainly exhibiting an inverse parabolic growth trend.

In terms of the network nodes, i.e., the number of transportation node cities, both the AIR and HSR networks exhibit linear growth trends, with the average growth rate of HSR node cities being about 4 times that of the AIR network, and the number of HSR node cities exceeding that of the AIR network after 2016. In terms of the network edges, i.e., the number of city pairs with direct transportation routes, the AIR network exhibits a linear growth trend, while the HSR network exhibits an inverse parabolic growth trend, and the number of city pairs with direct HSR services has exceeded the number of city pairs with direct air services since 2013. In terms of the network edge weights, i.e., the transportation frequencies or train numbers, the AIR network overall exhibits a linear growth trend, while the HSR network exhibits an inverse parabolic growth trend, with the growth rate of HSR train numbers being higher than that of air flight numbers.

**Fig 4:**
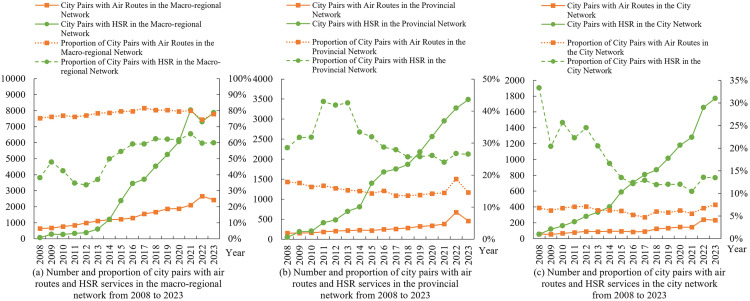
Trends of changes in the development scale of AIR and HSR networks at different dimensions from 2018 to 2023.

To further analyze the historical change trends of the distribution of the AIR and HSR networks at all dimensions nationwide, and due to the limitations of data availability, this study compares the number and proportion of city pairs with opened AIR routes and HSR train services at different dimensions, as shown in Fig 4. Overall, the proportion of city pairs with opened AIR and HSR services at the inter-macro-region dimension is on an upward trend, while the proportions at the inter-provincial within macro-regions and inter-city within provinces dimensions are both on a downward trend.

From the inter-macro-region dimension, the proportion of cross-macro-region city pairs in the AIR network is higher than that in the HSR network, maintaining around 80%, while the growth rate of the proportion of cross-macro-region city pairs in the HSR network is faster, reaching 60% by 2023. From the inter-provincial within macro-regions dimension, the proportion of cross-provincial city pairs in the HSR network is higher than that in the AIR network, and both are on a downward trend, with the HSR network declining faster. By 2023, the proportion of cross-provincial city pairs in the AIR network within macro-regions was 15%, while the proportion in the HSR network was 27%. From the inter-city within provinces dimension, the change trends of the AIR and HSR networks are similar to the inter-provincial within macro-regions dimension, but the proportions are lower, with the proportion of intra-provincial city pairs in the AIR network reaching 8% and in the HSR network reaching 13% by 2023.

#### 5.1.2. Spatial variation characteristics of the development scale of the HSR network and the AIR network.

**Fig 5:**
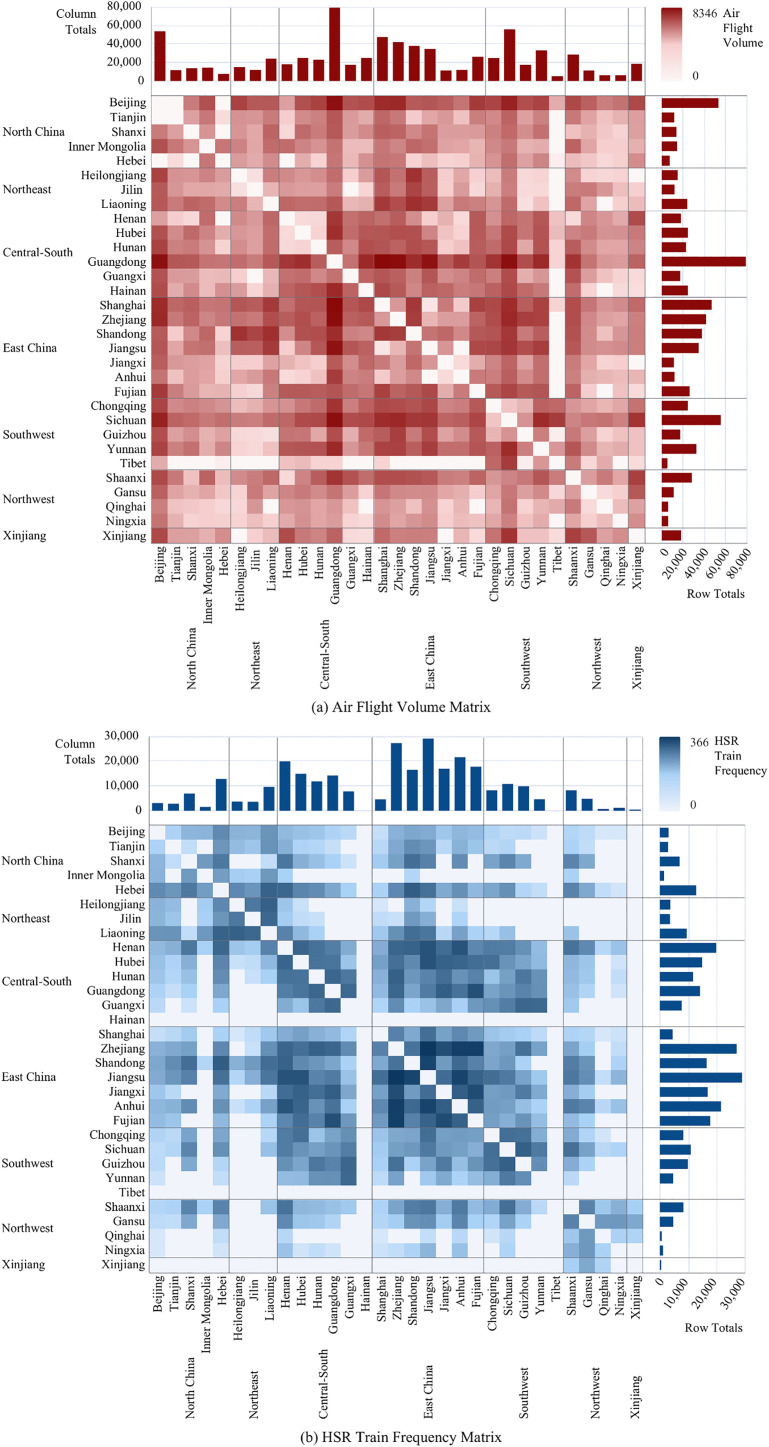
The spatial matrix of AIR flight volumes and HSR train frequency in China.

This study uses the distribution data of city pairs with opened AIR routes and HSR train services to compare the spatial distribution of transportation node cities and transportation routes in China’s AIR and HSR networks, as shown in Fig 5. Overall, the HSR train numbers and AIR flights exhibit spatial heterogeneity in terms of distribution balance, and there are obvious differences in the distribution characteristics within and across macro-regions.

From the geographic distribution perspective, in terms of provinces, the distribution of AIR flights is more balanced across the country compared to the HSR network, especially having extensive connections with the Southwest, Northwest, and Northeast provinces. However, in the densely populated North, Central-South, and East regions, the provinces with higher flight volumes are more concentrated in Beijing, Shanghai, Guangdong, and Sichuan, with large gaps compared to other provinces. The distribution of the HSR network is less balanced across the country, with HSR train numbers mainly concentrated in the Central-South and East regions, with large gaps between other provinces and these two regions, but smaller gaps between provinces within the Central-South and East regions, more balanced than the AIR network. In terms of macro-regions, the distribution of AIR flights is more concentrated across macro-regions than within macro-regions, with a concentration in the flights across the East, Central-South, Southwest, and North macro-regions, while the HSR train numbers are more clearly concentrated within the macro-regions, especially within the East and Central-South macro-regions.

From the network density perspective, the HSR network in the region south of the “Hu Line” has significantly higher numbers of transportation hubs, transportation route density, and spatial balance compared to the AIR network, while in the region north of the “Hu Line”, the AIR network has a much wider distribution of transportation nodes and routes compared to the HSR network.

Further analysis reveals the following characteristics and differences in the structure of the HSR network and the AIR network:

First, there are significant differences in the spatial distribution of the regional spatial connection networks, with the geographic distribution center of the AIR network being more westward and northward compared to the HSR network. As shown in Fig 5, the AIR network has more spatial connections involving the Northwest, Southwest, and Northeast regions, especially forming extensive connections between the central-eastern regions and Xinjiang province, while the HSR network has stronger internal connections within the Central-South, North, and East regions, and weaker connections with the Southwest, Northwest, and Northeast regions.

Second, the scope of the network-based connection pattern centered on hub cities has expanded, and the AIR network has stronger core region clustering characteristics compared to the HSR network, and correspondingly lower spatial balance. Under the influence of the AIR and HSR networks, the network-based economic connections centered on central cities have continuously expanded across the country, overall presenting a network-based spatial pattern centered on hub cities. However, there are differences between the AIR and HSR networks in the degree of promoting core region clustering, with the AIR network having a higher proportion of connections between provincial capital cities, mainly due to the stronger core region clustering characteristics of the AIR network in the Southwest, Central-South, and East regions compared to the HSR network.

### 5.2. Comparison of the structural quality of the HSR network and the AIR network

#### 5.2.1. Temporal variation characteristics of the structural quality of HSR and AIR networks.

**Fig 6:**
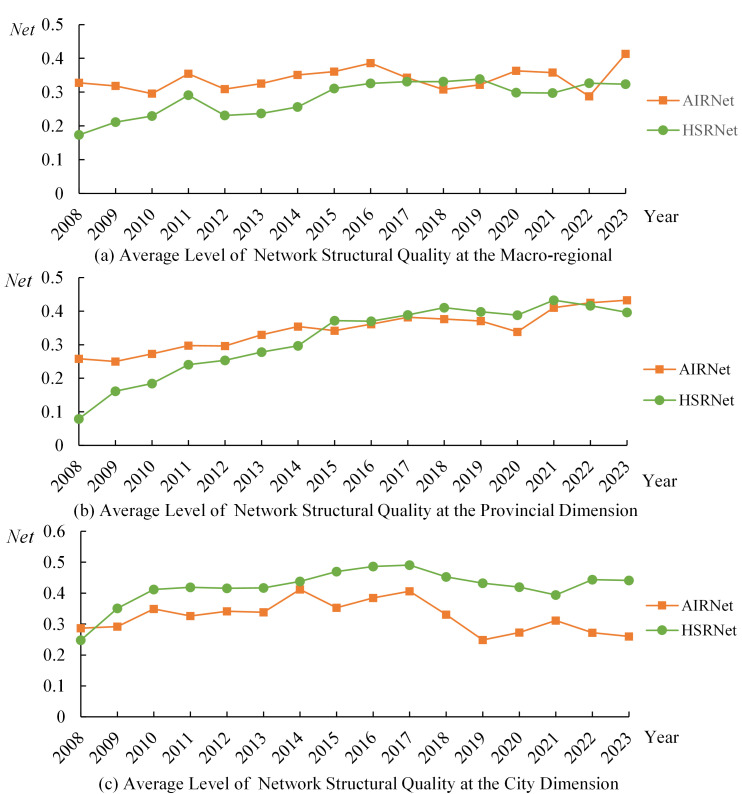
Trends of changes in the average levels of the structural quality of HSR and AIR networks in China at different dimensions, 2008−2023.

This study uses the constructed comprehensive index Net to measure the quality of the HSR and AIR networks at different dimensions in China, as shown in Fig 6. Overall, during 2008–2023, the structural quality of both HSR and AIR networks across all spatial scales in China exhibited a fluctuating upward trend. AIR networks predominated at the macro-regional scale, while HSR networks held advantages at the provincial and city scales. At the macro-regional scale, AIR network quality surpassed that of HSR during most periods. HSR briefly led in 2018–2019 and 2022, though AIR’s superiority expanded by 2023. At the provincial scale, AIR and HSR networks demonstrated comparable levels and trends; HSR held a marginal advantage post-2015, shifting to AIR after 2022. Notably, provincial-level networks exhibited the highest structural quality and fastest growth rates across all scales. At the city scale, HSR maintained a significant long-term advantage. However, declining HSR quality alongside rising AIR quality since 2019 narrowed this gap until 2021, after which the disparity reversed to expand.

#### 5.2.2. Spatial variation characteristics of the structural quality of the HSR network and the AIR network.

**Fig 7:**
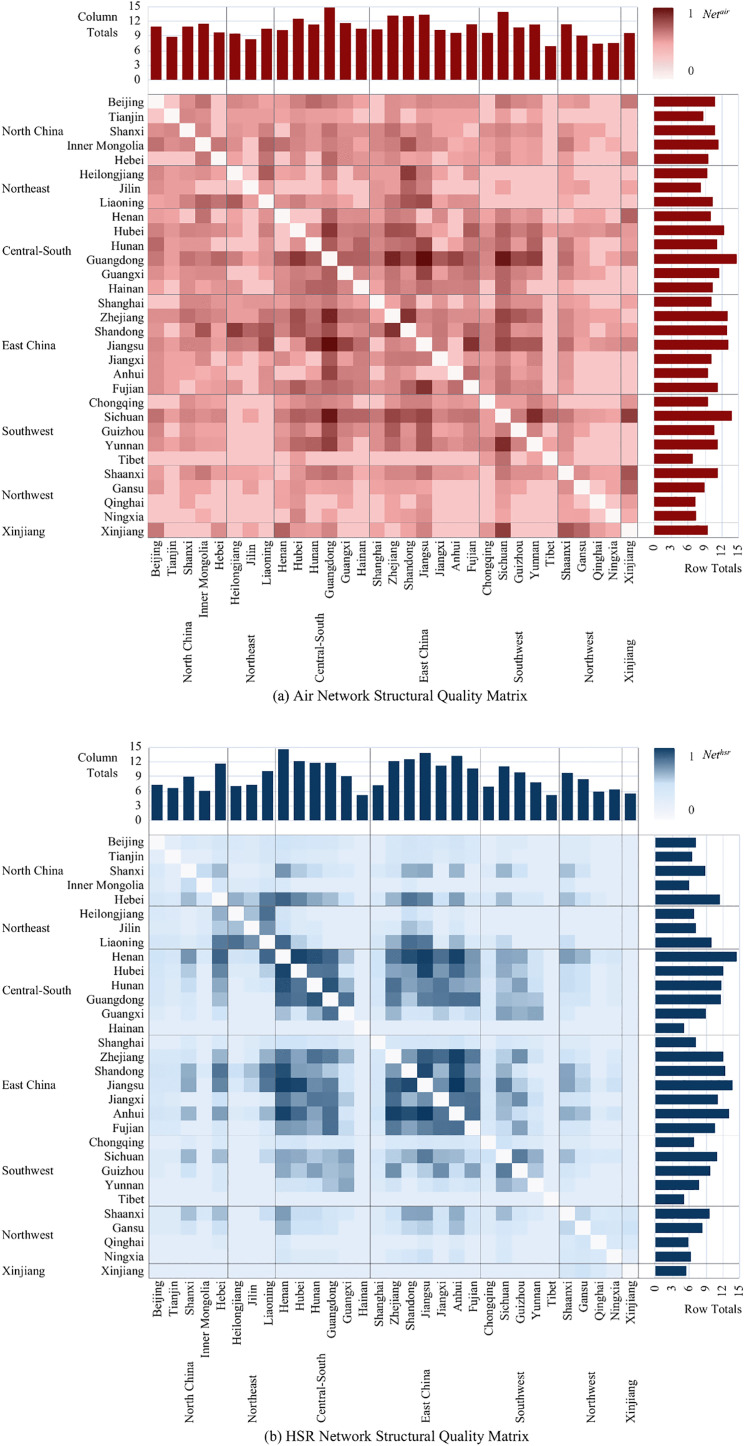
The spatial matrix of the structural quality of the AIR and HSR networks in China.

To analyze the spatial differentiation of the structural quality of the HSR and AIR networks, this study constructed a spatial matrix based on the provincial-level structural quality indicators of the AIR and HSR networks, as shown in Fig 7. Overall, the HSR and AIR networks exhibit obvious differences in distribution balance, as well as in the distribution characteristics within and across macro-regions.

From the provincial perspective, the structural quality of the AIR network formed between provinces is more balanced than that of the HSR network. In the AIR network, except for the relatively low quality in Tibet, Qinghai, and Ningxia provinces, the structural quality of the AIR network formed between other provinces is not significantly different, indicating a balanced development of the AIR network structure across the country. In the HSR network, the structural quality of the HSR network formed between provinces in the Central-South and East regions is significantly higher than other regions, with Henan, Jiangsu, and Anhui provinces having the highest quality compared to other provinces, indicating that the balanced development of the HSR network structure across the country is not as good as the AIR network.

From the macro-regional perspective, the structural quality of the AIR network formed between provinces across macro-regions is higher than that formed between provinces within macro-regions, with the quality being relatively high in the connections across the East, Central-South, Southwest, and North macro-regions. In contrast, the structural quality of the HSR network is higher within the macro-regions, mainly reflected in the East and Central-South macro-regions.

The above descriptive statistics and discussions help us to comprehensively understand the differences between the HSR network and the AIR network, as well as their potential heterogeneous impacts on CRED. Through the above analysis, we believe that the HSR network and the AIR network exhibit dimensional heterogeneity in promoting CRED, with the HSR network being more capable of promoting CRED at the city and provincial dimensions, while the AIR network is more capable of promoting CRED at the macro-regional dimension. However, the observed patterns may be confounded by many factors specific to certain regions, and we have also found some factors in the HSR network and AIR network that may inhibit CRED.

Furthermore, it is difficult to precisely separate the macro-regional, provincial, and city-level networks through intuitive observation. Therefore, in Section 6, we will use econometric estimation to conduct more rigorous empirical testing.

## 6. Empirical testing

### 6.1. Overall baseline regression based on network dimensions

We conduct the baseline regression, and the estimation results are shown in Table 2. Columns (1) to (3) present the regression results for the macro-regional network, Columns (4) to (6) present the results for the provincial network, and Columns (7) to (9) present the results for the city network. In the testing process for the networks at each dimension, we separately verify the impacts of AIR, HSR, and HSR-AIR collaboration (HA) on coordinated regional economic development (CRED), and the results show that the impacts of the above three factors on CRED differ greatly across dimensions.

**Table 2:** Baseline regression results of networks at different dimensions based on coordinated regional economic development.

	(1)	(2)	(3)	(4)	(5)	(6)	(7)	(8)	(9)
Dependent Variable	*CRED*	*CRED*	*CRED*	*CRED*	*CRED*	*CRED*	*CRED*	*CRED*	*CRED*
Network Dimension	Macro-regional Network	Macro-regional Network	Macro-regional Network	Provincial Network	Provincial Network	Provincial Network	City Network	City Network	City Network
*AIR*	**0.163** ^***^		**0.121** ^***^	0.016		0.010	−0.010^*^		−0.009^*^
	**(0.040)**		**(0.036)**	(0.012)		(0.012)	(0.005)		(0.005)
*HSR*		0.031	−0.132		0.006	0.001		**0.025** ^***^	**0.025** ^***^
		(0.018)	(0.122)		(0.007)	(0.006)		**(0.008)**	**(0.008)**
*HA*			0.142			**0.024** ^***^			−0.002
			(0.104)			**(0.007)**			(0.002)
Control Variables	Yes	Yes	Yes	Yes	Yes	Yes	Yes	Yes	Yes
Regional Pair Fixed Effects	Yes	Yes	Yes	Yes	Yes	Yes	Yes	Yes	Yes
Year Fixed Effects	Yes	Yes	Yes	Yes	Yes	Yes	Yes	Yes	Yes
*N*	273	273	273	845	845	845	33020	33020	33020
*R* ^*2*^	0.981	0.980	0.981	0.996	0.996	0.996	0.903	0.903	0.903
adj. *R*^*2*^	0.977	0.977	0.978	0.995	0.995	0.995	0.895	0.895	0.895

Note: ^*^, ^**^, and ^***^ represent significance levels of 10%, 5%, and 1%, respectively, with clustered standard errors in parentheses, which are the same for the following tables.

Columns (1) to (3) in Table 2 present the baseline regression results for the impacts of AIR, HSR, and HA on CRED at the macro-regional scale. The results indicate that the AIR coefficient is statistically significant and positive at the 1% level. Since all variables in this study have been standardized, the regression coefficients can be compared horizontally to determine the impact of the standard deviation change of each variable on the standard deviation change of CRED. Comparing the coefficient values, the promotion effect of AIR on CRED at the macro-regional dimension is the strongest, followed by HA and HSR, indicating that promoting the development of the AIR network is the top priority for improving economic coordinated development between macro-regions.

Columns (4) to (6) in Table 2 report the baseline regression results at the provincial dimension. The regression results show that the estimated coefficients of HA are significantly positive at the 1% level, indicating that the development of the HSR-AIR collaboration have significantly improved the economic coordinated development at the provincial dimension. Comparing the coefficient values, HA has a relatively strong promotion effect, indicating that developing air-rail intermodal transport represents the primary strategy for enhancing provincial-level economic coordination within the transportation sector.

Columns (7) to (9) in Table 2 report the baseline regression results at the city dimension. The regression results show that HSR is significantly positive at the 1% level, indicating that the development of the HSR network significantly improves the economic coordinated development at the city dimension, and promoting the development of the HSR network is the top priority for promoting economic coordinated development at the city dimension. The collaboration between HSR and AIR within the same province has an inhibiting effect on economic coordinated development, which may be due to the relatively close geographical distance and high degree of industrial and downstream market overlaps between cities within the same province, leading to the problem of using non-market means to engage in malicious competition [93]. Furthermore, due to the closer geographical distance between cities within the same province compared to the other two dimensions, the mobility costs of labor, capital, education, and medical resources are lower, and the current profound changes in the spatial structure of China’s economic development have led to central cities and urban agglomerations becoming the main spatial forms to accommodate development factors, resulting in a more serious suction effect at the city dimension [25].

### 6.2. Dose-response function

#### 6.2.1. Macro-regional network.

This study uses the DR model to estimate the DRF of the impacts of HSR, AIR, and HSR-AIR collaboration (HA) on coordinated regional economic development (CRED). In the DR model used, the identification of the treatment and control groups and the selection of variables are consistent with the models shown in Equations (12),(13), and (14). Following previous studies, the polynomial order of the DRF is set to 3 [90].

**Fig 8:**
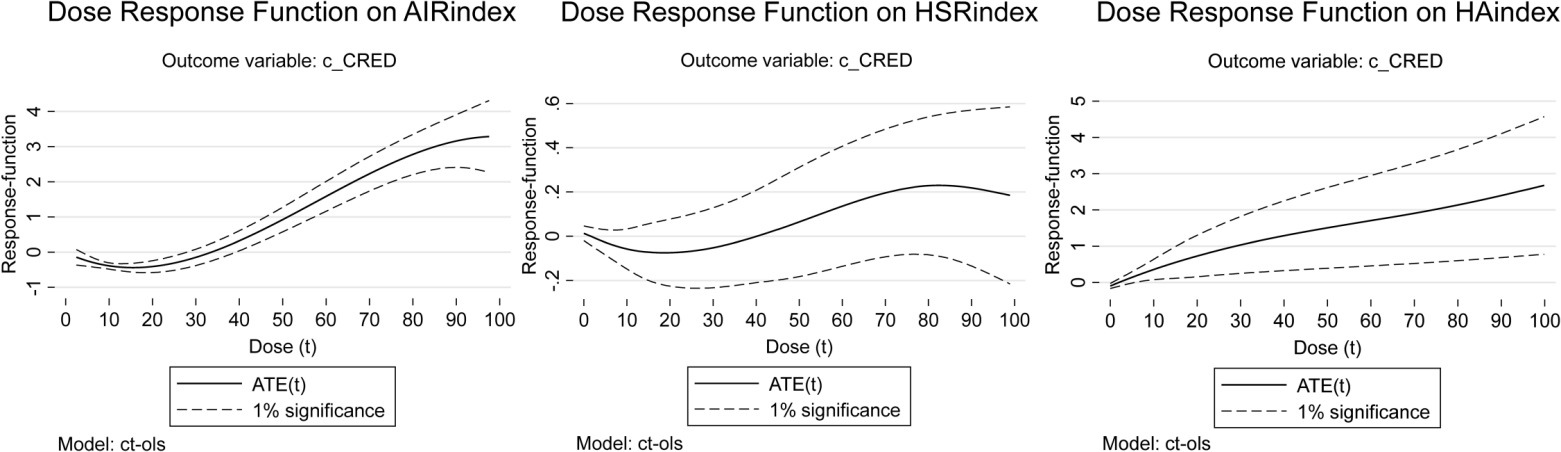
Application of the macro-regional network to examine the DRF of the impacts of AIR, HSR, and HSR-AIR collaboration (HA) on coordinated regional economic development (CRED).

Fig 8 shows that the overall impact of AIR, HSR, and HA on CRED in the macro-regional network increases as they develop, but the fluctuation trends of the three explanatory variables are different. The impacts of AIR and HA on CRED exhibit an upward trend as AIR and HA develop, with the confidence interval of AIR being narrower than that of HA, indicating that the impact of AIR is more robust. Thus, AIR consistently represents the optimal choice within rapid transport networks for promoting CRED at the macro-regional scale.

Based on the above results, it can be seen that the promotion effect of AIR on CRED is relatively stable and significant, which may be due to the technological advantages of air transportation, such as high speed and wide coverage, which are more effective at the macro-regional dimension and are conducive to continuously enhancing the connection strength between regions. However, the impact of HSR exhibits a certain critical value and lag effect, and excessive investment may lead to distortions in resource allocation, resulting in negative impacts in certain developmental stages. The impact of HA on CRED also exhibits a continuous improvement trend, indicating that the coordinated development of HSR and AIR can fully leverage their respective technological advantages and produce synergistic effects, thereby better promoting the coordinated regional economic development. This may also reflect the differences in regional development stages, i.e., in more economically mature regions, AIR may become the optimal choice, while in less developed regions, HA may have a greater promotion effect.

#### 6.2.2. Provincial network.

**Fig 9:**
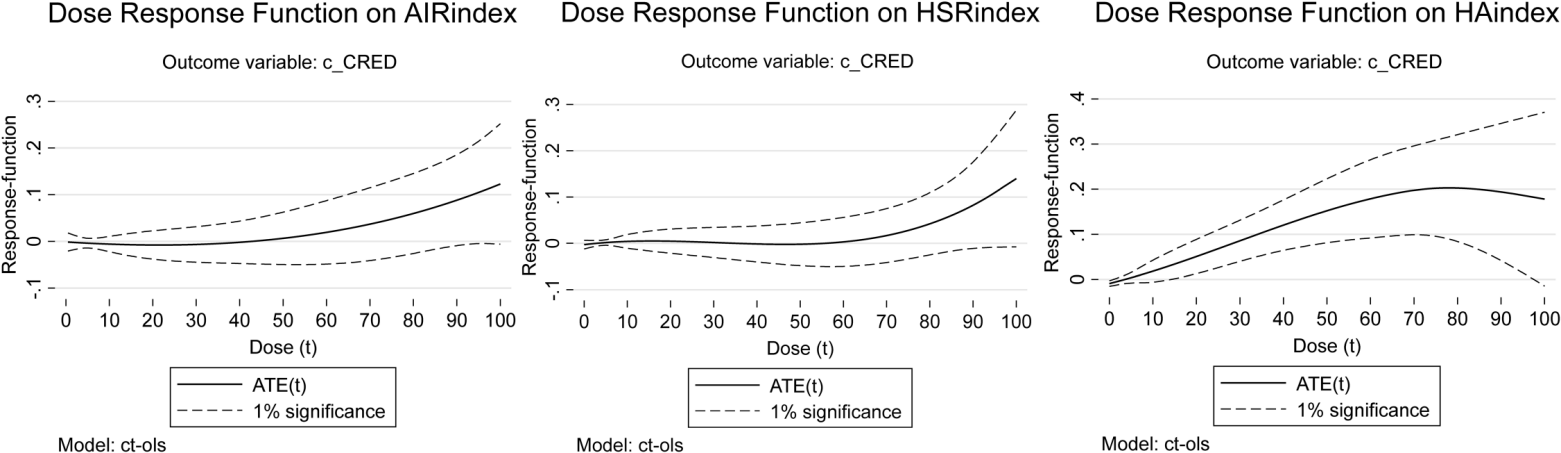
Application of the provincial network to examine the DRF of the impacts of AIR, HSR, and HSR-AIR collaboration (HA) on coordinated regional economic development (CRED).

Fig 9 shows the trends of the impacts on coordinated regional economic development (CRED) in the provincial network as AIR, HSR, and HSR-AIR collaboration (HA) develop. At the provincial dimension, the impact of HA on CRED exhibits an overall upward trend as HA develops, with the confidence interval of HA being at a significant level in the (10, 90) interval, with the coefficient peaking within the (70, 80) range and subsequently stabilizing. These findings indicate that enhancing transport service levels overall through HSR-AIR integration is more effective for promoting provincial CRED. However, this effect encounters an upper effectiveness threshold; for province pairs with higher baseline CRED levels, HA’s impact cannot be further augmented.

Furthermore, AIR and HSR individually demonstrate no statistically significant impact on CRED at the provincial scale, underscoring the necessity of developing HSR-AIR cooperation and advancing air-rail intermodal transport to leverage their combined potential for CRED promotion. Thus, while AIR consistently serves as an effective promoter of CRED at the broader macro-regional scale, reliance on any single transport mode proves limited at the more granular provincial level. This divergence likely arises from heterogeneous regional development patterns across spatial scales. Macro-regional transport networks may better encompass broader economic linkages, whereas provincial implementation necessitates accommodating distinct developmental stages and localized demands. In this context, the complementary technical and economic attributes of different transport modes enable synergistic development, maximizing their respective advantages and generating cooperative effects that more effectively foster regional economic coordination.

#### 6.2.3. City network.

**Fig 10:**
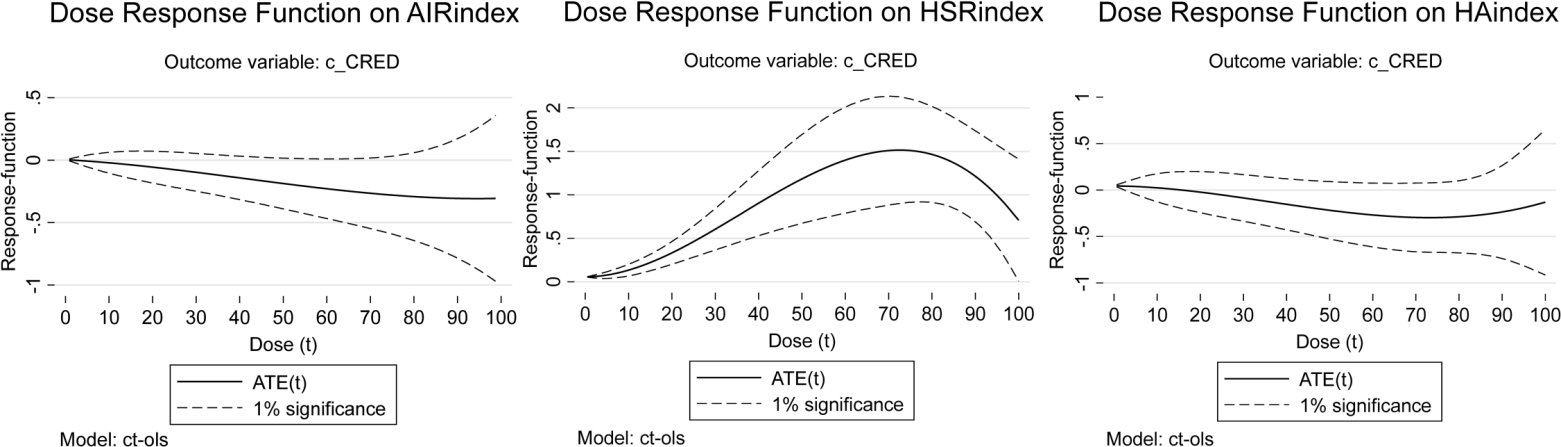
Application of the city network to examine the DRF of the impacts of AIR, HSR, and HSR-AIR collaboration (HA) on coordinated regional economic development (CRED).

Fig 10 shows the trends of the impacts on coordinated regional economic development (CRED) in the city network as AIR, HSR, and HSR-AIR collaboration (HA) develop. At the city scale, HSR maintains a significantly positive impact on CRED*,* with its effect initially rising then declining alongside HSR development; an inflection point occurs within the (70, 80) range. This result indicates that HSR is the best choice for promoting CRED at the city dimension, and its promotion effect strengthens as HSR develops, but the promotion effect declines in the cities where the HSR network development level is in the middle to upper range. This suggests that in the cities with the largest HSR network scale, the promotion effect of HSR on CRED exhibits diminishing marginal returns and cannot continuously drive CRED. This indicates that for regions with relatively high levels of economic coordinated development, relying solely on the improvement of transportation infrastructure may no longer achieve significant effects, and it is also necessary to promote regional economic coordination through the optimization of industrial structure and factor mobility. This may also be because the coordinated development level between the cities with the strongest transportation connections has already reached a relatively high level, and no further promotion by transportation is needed.

Furthermore, at the city dimension, the impacts of AIR and HA on CRED are always not significant, indicating that AIR is unable to play a role in promoting CRED at the city dimension. This is due to the transportation characteristics of AIR, which in the city dimension cannot leverage the advantage of fast transportation over long distances. Therefore, relying solely on AIR is difficult to effectively promote CRED, indicating that the selection of transportation modes should be tailored to local conditions and fully consider the actual needs at different spatial levels.

### 6.3. Robustness checks

#### 6.3.1. Endogeneity test.

This study examines the impacts of the HSR network and the AIR network on coordinated regional economic development (CRED) and has made efforts to make the explanatory variables and control variables as comprehensive and exogenous as possible. However, due to the following reasons, the reliability of the baseline regression results may still be affected by endogeneity issues:

① Omitted variable bias: The level of economic coordinated development between regional pairs is influenced by the overall macroeconomic system, and the development of regional transportation is also closely related to the macroeconomic environment, so there may be other unobservable factors that affect both the core explanatory variables and the dependent variable, causing a correlation between the core explanatory variables and the random error term, such as certain industrial policies or subsidy policies that may have simultaneously improved the development level of regional transportation and economic coordinated development [94].
② Measurement error: The improvement in the development level of regional transportation networks may not only be reflected in the network scale and network structure, but also in the geographic location of the network, and the description of the development level of network structural quality may also be reflected in some more complex aspects [95], so the indicators constructed in this study may have certain limitations in their explanatory power for the development level of the HSR network and the AIR network.
③ Reverse causality: CRED may have an economic supporting role for the development of regional transportation, providing an important economic foundation for the construction of transportation infrastructure such as HSR, which may lead to reverse causality problems [94]. Therefore, this study uses the instrumental variable method to mitigate the potential endogeneity problems mentioned above.

Following the research approach of Shen (2023) [96], this study uses the Bartik method to construct the instrumental variables $Scale\_IV^{t,air}$, $Scale\_IV^{t,hsr}$, $Net\_IV^{t,air}$, and $Net\_IV^{t,hsr}$. Based on this, the instrumental variables for the core independent variables, including $AIR\_IV_{pq}^{t}$, $HSR\_IV_{pq}^{t}$, and $HA\_IV_{pq}^{t}$ are calculated and incorporated into the model for parameter estimation, with the calculation formulas as follows.


$$\begin{array}{c} X\_IV_{pq}^{t,r}=X_{pq}^{t,r}\times \left(\frac{\sum {\mathrm{\backslash nolimits}}_{PQ\neq pq}X_{pq}^{t,r}}{\sum {\mathrm{\backslash nolimits}}_{PQ\neq pq}X_{pq}^{t-1,r}}\right)\\ X\_IV_{pq}^{t,r}=Scale\_IV_{pq}^{t,air},Scale\_IV_{pq}^{t,hsr},Net\_IV_{pq}^{t,air},Net\_IV_{pq}^{t,hsr} \end{array}$$ (21)

$$\begin{array}{c} AIR\_IV_{pq}^{t}=\frac{\text{1}}{\text{2}}\left(Scale\_IV_{pq}^{t,air}+Net\_IV_{pq}^{t,air}\right)\\ HSR\_IV_{pq}^{t}=\frac{\text{1}}{\text{2}}\left(Scale\_IV_{pq}^{t,hsr}+Net\_IV_{pq}^{t,hsr}\right)\\ HA\_IV_{pq}^{t}=AIR\_IV_{pq}^{t}\times HSR\_IV_{pq}^{t} \end{array}$$ (22)

Where *i* and *j* represent two regions without distinction of direction, and *t* represents the year. $X\_IV_{pq}^{t,r}$ represents the estimated value of the regional transportation network scale or structural quality, and $X_{pq}^{t-1,r}$ represents the original value of the regional transportation network scale or structural quality in the previous year. The indicators in the brackets represent the overall development speed of the regional transportation network scale or structural quality in year *t*. The overall development speed for each regional pair in each year needs to be calculated separately, and the AIR or HSR business volume of the region itself is not included, in order to ensure that the newly generated instrumental variables meet both the relevance and exogeneity conditions, and the calculations are carried out separately at the macro-regional, provincial, and city dimensions. In addition to the above constructed variables, this study also introduces the lagged terms of the core independent variables as a second set of instrumental variables.

The Bartik IV leverages exogenous variation from the overall development of the transportation network, excluding the region’s own business volume. This ensures relevance, as overall network development is correlated with regional network development. For exogeneity, by excluding internal business volume, the IV captures external shocks that are unlikely to directly affect CRED through channels other than the transportation network, thus satisfying the exclusion restriction.

Table 3 presents the results of the instrumental variable tests at the macro-regional, provincial, and city dimensions, respectively. The estimation method used is the two-stage least squares (2SLS) method, which is the standard practice in the instrumental variable approach and can effectively address endogeneity issues. To ensure the validity of the instrumental variables, the over-identification test, weak instrument test, and overidentification test were conducted. The data in the three tables show that the KP-LM statistic is significant, the KP-F statistic and CD-F statistic are both greater than the 10% bias critical value, and the Hansen J statistic p-value is greater than 0.1, indicating that the instrumental variables selected in this study have passed the over-identification test, weak instrument test, and over-identification test. Further comparison of the estimated coefficients of the core independent variables in the table reveals that they are relatively close to the baseline regression results, suggesting that the impact of endogeneity on the estimation results is small, and the baseline regression results in this section are robust.

**Table 3:** Estimation results of the instrumental variables.

	(1)	(2)	(3)	(4)	(5)	(6)	(7)	(8)	(9)
Dependent Variable	*CRED*	*CRED*	*CRED*	*CRED*	*CRED*	*CRED*	*CRED*	*CRED*	*CRED*
Network Dimension	Macro-regional Network	Macro-regional Network	Macro-regional Network	Provincial Network	Provincial Network	Provincial Network	City Network	City Network	City Network
*AIR*	0.255^**^		0.216^**^	0.005		−0.005	−0.003		−0.002
	(0.103)		(0.103)	(0.013)		(0.014)	(0.005)		(0.005)
*HSR*		0.030	−0.187		0.003	0.004		0.016^**^	0.016^**^
		(0.022)	(0.128)		(0.005)	(0.005)		(0.006)	(0.007)
*HA*			0.191^*^			0.011^**^			−0.004^*^
			(0.109)			(0.005)			(0.002)
Control Variables	Yes	Yes	Yes	Yes	Yes	Yes	Yes	Yes	Yes
Fixed Effects	Yes	Yes	Yes	Yes	Yes	Yes	Yes	Yes	Yes
KP-LM statistic	8.447^**^	5.079^*^	12.675^**^	12.991^***^	10.630^***^	18.536^**^	20.094^***^	30.044^**^	9.438^*^
KP-F statistic	65.198	1976.593	22.430	125.557	653.970	48.051	336.564	139.348	20.693
CD-F statistic	178.025	1449.536	53.828	712.877	1828.830	146.628	23000	26000	3530.490
10% bias critical value	19.930	19.930	12.200	19.930	19.930	12.200	19.930	19.930	12.200
Hansen J statistic p-value	0.897	0.666	0.798	0.1223	0.8345	0.162	0.382	0.136	0.281

The comparison between the OLS (Table 2) and IV estimates (Table 3) reveals that the latter are generally larger in magnitude and statistical significance across key dimensions. This pattern, where IV estimates exceed their OLS counterparts, is not only plausible but provides critical insights into the nature of the endogeneity biases present in our model. We posit that the dominant source of bias is a downward bias in the OLS estimates, primarily driven by measurement error and reinforced by specific forms of omitted variable bias.

The first is the attenuation bias from measurement error. The constructions of HSR and AIR network development are inherently complex. Our measures of network scale and structure, while comprehensive, are inevitably imperfect proxies for the true, underlying connectivity and quality of service. For instance, our data on flight and train frequencies may not fully capture load factors, actual passenger volumes, or the qualitative aspects of service (e.g., comfort, reliability). Such non-classical measurement error in the explanatory variables is well-established in econometric theory to cause attenuation bias, i.e., a bias of the OLS coefficients toward zero [97]. The IV strategy, by leveraging exogenous variation from the Bartik-type instruments that are less correlated with these measurement errors, corrects for this attenuation, thereby recovering larger and likely more accurate estimates of the true causal effect.

The second is the bias of omitting variable bias from constraining policies. A common concern is that omitted variables, such as unobserved regional economic vitality, would positively correlate with both transport development and CRED, leading to an upward bias in OLS. However, the observed downward bias suggests the presence of omitted factors that are negatively correlated with our explanatory variables. In the Chinese context, a plausible candidate is the presence of local protectionist policies or administrative barriers. Regional governments might strategically restrain the outward expansion of their transportation networks to protect local industries or retain economic resources within their jurisdiction, or they might face regulatory hurdles in integrating with neighboring regions. These unobserved institutional and political factors could simultaneously inhibit the full development of inter-regional transport networks (a negative effect on HSR/AIR) and hinder economic coordination (a negative effect on CRED). The omission of such “connectivity-constraining” factors from the OLS model would introduce a negative correlation between the error term and our key variables, biasing the OLS estimates downward. Our IV, which is based on nationwide network growth trends exogenous to any specific regional pair’s political economy, helps to purge this bias.

The final point is the role of reverse causality. While reverse causality, where higher CRED leads to more transport investment, could theoretically cause an upward bias, its net effect in our setting may be muted or dominated by the aforementioned forces. The planning and construction of large-scale transport infrastructure like HSR and major air routes in China are often determined by central government strategies and long-term plans, which may not instantaneously respond to short-to-medium term fluctuations in regional economic coordination. Furthermore, if better-coordinated regions engage in more efficient, demand-driven infrastructure planning that avoids redundant investment, it could even lead to a negative correlation. Therefore, the net effect of reverse causality is ambiguous but is unlikely to generate a strong upward bias that would counteract the clear downward pressure from measurement error and constraining policies.

In conclusion, the finding that the IV estimates are larger than the OLS estimates is economically meaningful and methodologically reassuring. It is consistent with a model where the “true” positive effect of transportation networks on CRED is partially obscured in OLS regressions by measurement inaccuracies and unobserved institutional barriers to integration. The IV approach, by addressing these sources of endogeneity, provides a more credible and likely less biased estimation of the net causal impacts.

#### 6.3.2. Shortening the time period.

The sample period selected in this study is from 2008 to 2021. In the robustness check, we further consider the impact of global macroeconomic events. The global financial crisis that occurred in 2008 had a lasting impact on the global economy until 2010 [98], and the COVID-19 pandemic that began to spread globally at the end of 2019 has had an impact on the global economy since 2020. Therefore, this study shortens the sample period and uses panel data from 2011 to 2019 to re-conduct the baseline regression, in order to test the robustness of the empirical results. The regression results are shown in Table 4, which indicate that the estimated coefficients of the core explanatory variables are close to the baseline regression results, further confirming the overall robustness of the conclusions in this section.

**Table 4:** Estimation results with shortened sample period.

	(1)	(2)	(3)	(4)	(5)	(6)	(7)	(8)	(9)
Dependent Variable	*CRED*	*CRED*	*CRED*	*CRED*	*CRED*	*CRED*	*CRED*	*CRED*	*CRED*
Network Dimension	Macro-regional Network	Macro-regional Network	Macro-regional Network	Provincial Network	Provincial Network	Provincial Network	City Network	City Network	City Network
*AIR*	0.274^***^		0.219^**^	−0.002		−0.00002	0.00005		0.0005
	(0.083)		(0.084)	(0.011)		(0.011)	(0.005)		(0.004)
*HSR*		0.034	−0.131		0.008^*^	0.005		0.010^**^	0.010^**^
		(0.020)	(0.104)		(0.004)	(0.004)		(0.004)	(0.005)
*HA*			0.140			0.009^**^			−0.003^**^
			(0.090)			(0.004)			(0.001)
Control Variables	Yes	Yes	Yes	Yes	Yes	Yes	Yes	Yes	Yes
Fixed Effects	Yes	Yes	Yes	Yes	Yes	Yes	Yes	Yes	Yes
*N*	189	189	189	585	585	585	22860	22860	22860
*R* ^*2*^	0.989	0.989	0.990	0.999	0.999	0.999	0.953	0.953	0.953
adj. *R*^*2*^	0.987	0.986	0.987	0.999	0.999	0.999	0.947	0.947	0.947

#### 6.3.3. PPML model.

In earlier years (2014 and before), the development level of China’s HSR was still relatively low, and there were no HSR train services between many regional pairs, resulting in many zero values. At the city pair level, not all of the 2,540 city pairs in China have HSR train services and AIR flights, as many small and medium-sized cities usually transit through nearby larger cities to be included in the national high-speed transportation network. Therefore, this study re-estimates using the Pseudo-Poisson Maximum Likelihood (PPML) method, which has the advantage of consistently estimating even when the sample has a large proportion of zero values [96], and the results are shown in Table 5.

**Table 5:** Estimation results of the PPML model test.

	(1)	(2)	(3)	(4)	(5)	(6)	(7)	(8)	(9)
Dependent Variable	*CRED*	*CRED*	*CRED*	*CRED*	*CRED*	*CRED*	*CRED*	*CRED*	*CRED*
Network Dimension	Macro-regional Network	Macro-regional Network	Macro-regional Network	Provincial Network	Provincial Network	Provincial Network	City Network	City Network	City Network
*AIR*	0.197^***^		0.129^***^	0.045^*^		0.032	−0.003		−0.003
	(0.040)		(0.039)	(0.023)		(0.021)	(0.002)		(0.002)
*HSR*		0.039^**^	−0.082		0.006	0.007		0.005^***^	0.005^***^
		(0.019)	(0.108)		(0.009)	(0.009)		(0.001)	(0.001)
*HA*			0.093			0.046^***^			0.001
			(0.087)			(0.009)			(0.001)
Control Variables	Yes	Yes	Yes	Yes	Yes	Yes	Yes	Yes	Yes
Fixed Effects	Yes	Yes	Yes	Yes	Yes	Yes	Yes	Yes	Yes
*N*	273	273	273	845	845	845	33020	33020	33020

The regression results show that the estimated coefficients of the core explanatory variables are all significantly positive and close to the baseline regression, further confirming the robustness of the basic conclusions in this study.

#### 6.3.4. Variable winsorization.

In actual data, there are often some extreme outliers, such as the transportation infrastructure development level in some economically underdeveloped regions being far lower than the average level. These extreme values may cause the regression results to deviate from the true situation. This study effectively excludes the influence of extreme outliers on the regression results by winsorizing the variables at the 2% left tail, and the results are shown in Table 6. The regression results show that the estimated coefficients of the core explanatory variables are all significantly positive and close to the baseline regression, further confirming the robustness of the basic conclusions in this study.

**Table 6:** Estimation results of the variable winsorization test.

	(1)	(2)	(3)	(4)	(5)	(6)	(7)	(8)	(9)
Dependent Variable	*CRED*	*CRED*	*CRED*	*CRED*	*CRED*	*CRED*	*CRED*	*CRED*	*CRED*
Network Dimension	Macro-regional Network	Macro-regional Network	Macro-regional Network	Provincial Network	Provincial Network	Provincial Network	City Network	City Network	City Network
*AIR*	0.168^***^		0.128^***^	0.015		0.009	−0.010^*^		−0.009^*^
	(0.040)		(0.035)	(0.012)		(0.012)	(0.005)		(0.005)
*HSR*		0.032^*^	−0.126		0.006	0.001		0.025^***^	0.025^***^
		(0.018)	(0.123)		(0.007)	(0.006)		(0.008)	(0.008)
*HA*			0.136			0.024^***^			−0.002
			(0.105)			(0.007)			(0.002)
Control Variables	Yes	Yes	Yes	Yes	Yes	Yes	Yes	Yes	Yes
Fixed Effects	Yes	Yes	Yes	Yes	Yes	Yes	Yes	Yes	Yes
*N*	273	273	273	845	845	845	33020	33020	33020
*R* ^*2*^	0.981	0.980	0.982	0.996	0.996	0.996	0.902	0.902	0.902
adj. *R*^*2*^	0.978	0.977	0.978	0.995	0.995	0.995	0.893	0.894	0.894

## 7. Conclusions

This study explores the impacts of the two major high-speed transportation networks, HSR and AIR, on coordinated regional economic development (CRED), and examines the potential and effects of their coordinated development in promoting CRED. The main research conclusions are as follows:

First, this study provides comprehensive empirical evidence, detailing and analyzing the current levels of CRED, the development of the HSR network, and the development of the AIR network in China. The research results show that the current CRED level in China exhibits significant spatial unevenness, with the overall distribution following the pattern of “within macro-regions> between macro-regions” and “East China> North China and Central-South> others”. Both the AIR and HSR networks in China are continuously expanding, with the AIR network exhibiting a linear growth trend and the HSR network mainly exhibiting a parabolic growth trend. Divided by the “Heihe-Tengchong Line”, the HSR network is dense and balanced in the southeast, while the AIR network is concentrated in hubs, and the AIR network is more widely distributed in the northwest than the HSR network. The geographic distribution center of the AIR network is more westward and northward compared to the HSR network, and it has stronger core region clustering characteristics and lower balance accordingly. The conclusions of the descriptive analysis provide empirical evidence for the dimensional heterogeneity of HSR and AIR networks in promoting CRED.

Secondly, the overall conclusions of the empirical tests prove that there is a certain complementarity between HSR and AIR, and through coordinated development, they can produce synergistic effects, thereby better promoting regional economic coordination. Specifically, at the macro-regional dimension, AIR has the strongest and most significant promotion effect on CRED; at the provincial dimension, HSR-AIR collaboration is the most capable of promoting the development of CRED; while at the city dimension, the HSR network has the strongest ability to promote CRED. This conclusion supports the importance of formulating transportation network planning policies that are tailored to local conditions.

Finally, DR model results confirm that AIR networks exhibit the most pronounced promotive effect on CRED at the macro-regional scale, with stable enhancement alongside AIR development, which contributes to its high speed and extensive coverage. At the provincial scale, HSR-AIR collaboration development proves more critical for CRED, though this effect encounters an upper threshold, for province pairs with advanced intermodal collaboration levels, further enhancement becomes unattainable. Conversely, HSR emerges as the optimal facilitator for CRED at the city scale. Its promotive effect initially increases then decreases with HSR advancement, exhibiting diminishing marginal effects on CRED between moderately to highly developed HSR networks, thereby failing to sustain CRED momentum.

Based on the above research conclusions, we propose the following policy recommendations:

First, the government should fully consider the differential impacts of HSR and AIR networks on CRED at different spatial scales. At the macro-regional level, the development of a widely covered and highly connected AIR network should be prioritized to promote cross-regional economic connections and resource flows. At the inter-provincial level, the focus should be on building an efficient air-rail intermodal network, promoting the cooperation between HSR and AIR to enhance the coordinated development between provinces. At the city level, further efforts should be made to promote HSR infrastructure construction, leveraging its advantages in promoting economic coordination between cities. Such a hierarchical and tailored transportation network planning can help maximize the coordinated development effects of different networks at different spatial scales.

Secondly, the government should encourage the coordinated development of HSR and AIR networks and leverage their synergistic effects in promoting regional economic coordination. Measures such as optimizing hub station layouts and improving transit connections can enhance the interconnectivity between HSR and AIR, improving the level of transportation integration within and between regions. At the same time, attention should be paid to avoiding excessive competition at the city level, which can lead to resource waste and industrial structure homogenization, thereby affecting regional economic coordination.

Finally, the government should adopt differentiated policies based on the transportation network development levels of different regions. In areas with more developed transportation networks, the focus should be on optimizing and upgrading the HSR network; while in areas with relatively backward transportation networks, the priority should be on developing the AIR network to narrow regional development gaps. Meanwhile, close attention should be paid to changes in the marginal utility of transportation network development, and policy priorities should be adjusted in a timely manner to ensure that transportation infrastructure construction continues to play its due role in regional economic coordination.

## 8. Study limitations

While this study provides comprehensive insights into the comparative advantages of HSR and AIR networks in promoting CRED, several limitations should be acknowledged when interpreting our findings. First, the methodological approach of separating macro-regional, provincial, and city networks, while necessary for identifying scale-specific effects, might oversimplify the complex interactions between different spatial dimensions. In reality, these networks operate simultaneously and may exhibit cross-dimensional influences that our analytical framework cannot fully capture. Second, our measurement of transportation network development, while comprehensive in capturing scale and structural aspects, does not incorporate important qualitative dimensions such as service quality, pricing structures, and passenger comfort. These factors could significantly affect the actual utilization and economic impact of transportation networks. Third, regarding model specification, although the Dose-Response model represents an advancement over traditional approaches in handling heterogeneity, its parametric assumptions may not fully reflect the complex nonlinear relationships in the real world. The potential for residual endogeneity, despite our instrumental variable approach, also warrants caution. Finally, the exclusive focus on China’s context, while providing valuable insights for the world’s largest developing economy, limits the immediate generalizability of our findings to other institutional environments and developmental stages. Future research could benefit from comparative studies across different countries and regions. Notwithstanding these limitations, we believe our study makes meaningful contributions to understanding the scale-dependent effects of transportation networks on regional economic coordination. These limitations also present fruitful avenues for future research to build upon our findings.
